# Chitohexaose Activates Macrophages by Alternate Pathway through TLR4 and Blocks Endotoxemia

**DOI:** 10.1371/journal.ppat.1002717

**Published:** 2012-05-24

**Authors:** Santosh K. Panda, Sunil Kumar, Nitin C. Tupperwar, Tushar Vaidya, Anna George, Satyajit Rath, Vineeta Bal, Balachandran Ravindran

**Affiliations:** 1 Institute of Life Sciences, Bhubaneswar, India; 2 Centre for Cellular and Molecular Biology, Hyderabad, India; 3 National Institute of Immunology, New Delhi, India; NIAID/NIH, United States of America

## Abstract

Sepsis is a consequence of systemic bacterial infections leading to hyper activation of immune cells by bacterial products resulting in enhanced release of mediators of inflammation. Endotoxin (LPS) is a major component of the outer membrane of Gram negative bacteria and a critical factor in pathogenesis of sepsis. Development of antagonists that inhibit the storm of inflammatory molecules by blocking Toll like receptors (TLR) has been the main stay of research efforts. We report here that a filarial glycoprotein binds to murine macrophages and human monocytes through TLR4 and activates them through alternate pathway and in the process inhibits LPS mediated classical activation which leads to inflammation associated with endotoxemia. The active component of the nematode glycoprotein mediating alternate activation of macrophages was found to be a carbohydrate residue, Chitohexaose. Murine macrophages and human monocytes up regulated Arginase-1 and released high levels of IL-10 when incubated with chitohexaose. Macrophages of C3H/HeJ mice (non-responsive to LPS) failed to get activated by chitohexaose suggesting that a functional TLR4 is critical for alternate activation of macrophages also. Chitohexaose inhibited LPS induced production of inflammatory molecules TNF-α, IL-1β and IL-6 by macropahges *in vitro* and *in vivo* in mice. Intraperitoneal injection of chitohexaose completely protected mice against endotoxemia when challenged with a lethal dose of LPS. Furthermore, Chitohexaose was found to reverse LPS induced endotoxemia in mice even 6/24/48 hrs after its onset. Monocytes of subjects with active filarial infection displayed characteristic alternate activation markers and were refractory to LPS mediated inflammatory activation suggesting an interesting possibility of subjects with filarial infections being less prone to develop of endotoxemia. These observations that innate activation of alternate pathway of macrophages by chtx through TLR4 has offered novel opportunities to cell biologists to study two mutually exclusive activation pathways of macrophages being mediated through a single receptor.

## Author Summary

Sepsis is one of the leading causes of death contributing to mortality as high as 54 percent in intensive care units across the world. Hyper inflammation induced by bacteria or bacterial products through Toll like receptors leads to sepsis and hence current approaches are directed towards blockade such receptors. While many such candidate antagonists have shown promise they also result in induction of inappropriate innate immune responses thus increasing risk of development of shock leading to death. In this study we describe a novel approach to treat endotoxemia associated with sepsis, fundamentally different from other reports. Chitohexaose a small molecular weight polysaccharide by virtue of its ability to bind to active sites of TLR4 inhibited LPS induced production of inflammatory mediators by murine macrophages and human monocytes. Administration of chitohexaose with LPS blocked endotoxemia leading to mortality of mice. More significantly, Chitohexaose reversed inflammation and protected mice even 24/48 hrs after onset of endotoxemia. Apart from competitively inhibiting LPS induced inflammation chitohexaose also activated alternate pathway of macrophages. Such macrophages are known to display increased phagocytic activity, are resistant to LPS induced activation and associated with resolution of inflammation and tissue repair.

## Introduction

Sepsis and septic shock, one of the most common causes of admission in intensive care units results in death of nearly 3, 50,000 people every year only in US and Europe [1], [2]. The disease is a consequence of systemic bacterial infections that stimulates mediators of inflammation due to hyper activation of phagocytes. Immune cells express pattern recognition receptors (PRRs) which recognize immunostimulatory microbial products called PAMPs (pathogen associated molecular pattern) and trigger production of inflammatory mediators which assist the host in elimination of infectious agents [3], [4]; however hyper induction of such mediators by dysregulated innate immune cells leads to sepsis and septic shock [5], [6]. In sepsis caused by Gram-negative bacteria, endotoxin (LPS) activates the immune system through TLR4 and induces activation of macrophages that produce inflammatory mediators [7], [8]. TLR4 is the signaling receptor for LPS but doesn't directly bind to LPS [9]–[11]. LPS forms a complex with LPS binding protein and CD14 which in turn delivers LPS to MD2 and LPS-MD2 complex activates through TLR4 resulting in dimerization of TLR4 [3] and initiate the signaling process for production of cytokines and other critical molecules needed for hyper-inflammation associated with endotoxemia/sepsis [12].

While effective use of antibiotics has resulted in improved prognosis of sepsis, deterioration of clinical symptoms and mortality has been attributed to persistent inflammatory cascade. Neutralization of inflammation is considered essential for preventing severe consequences of sepsis [13], [14]. Thus developments of antagonists that block either activation through TLRs or downstream signaling pathways that inhibit the storm of inflammatory molecules are widely pursued by several investigators. Antibodies to TLR4 [15], [16], TLR4/MD2 [17] complex or LPS analogs [18], [19] have been tested in animal models for their efficacy to protect against enditoxemia/Gram-negative sepsis although only LPS analogues have been undergone clinical trials. Nitrate salts [20], 5c, an inhibitor of sphingosine kinase [21], oxidized phospholipid [22], [23] molecules have also offered promising results. Despite all these attempts very few candidate molecules have reached the level of clinical trials. A very recent report of one of the clinical trial for a promising agent was found to be ineffective (http://clinicaltrials.pharmaceutical-business-review.com/news/eisai-eritoran-fails-to-meet-primary-endpoint-in-phase-iii-trial-250111). In this study we report a novel mechanism that blocks endotoxemia by an approach fundamentally different from those documented so far. We demonstrate that a low molecular weight chito-oligosaccharide, chitohexaose (chtx) delicately balances the storm of inflammation induced by LPS while concurrently activating macrophages into a non inflammatory alternate pathway through TLR4. Administration of chtx protected mice from endotoxemia prophylactically as well as therapeutically. The study also offered evidence for induction of two diverse activation pathways of macrophages through a single receptor, TLR4. The stimulating ligand appears to determine the activation phenotype viz; classical pathway by LPS and alternate pathway by chtx. We stumbled on these findings while searching for the elusive innate receptors for nematodes.

## Results

### FAg binds to monocytes/macrophages through TLR4

**Figure 1:**
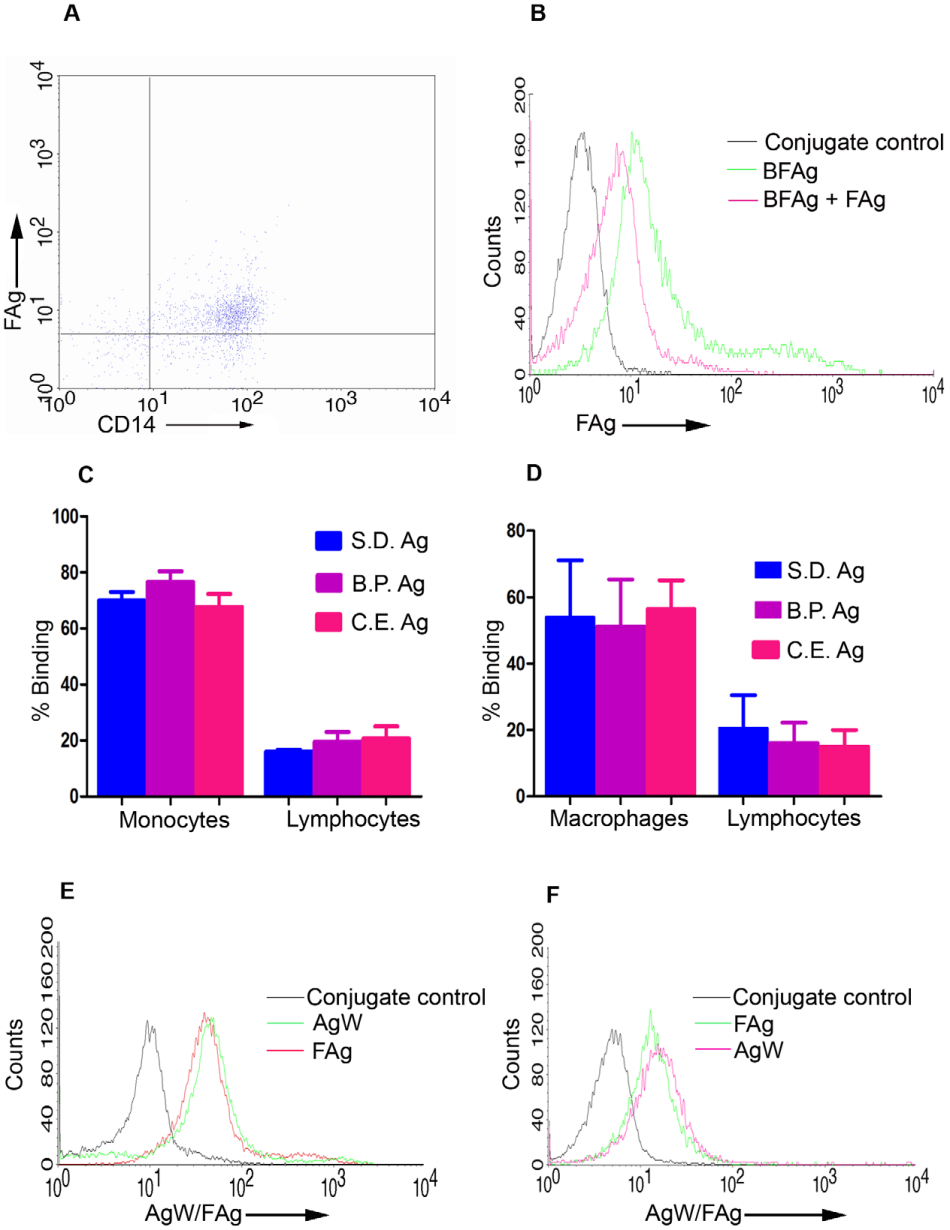
FAg/AgW binds to both human monocytes and murine bone marrow macrophages. (**A**) Human PBMCs were incubated with biotinylated FAg followed by staining with Streptavidin-PE and CD14 FITC. Streptavidin-PE as conjugate control and FITC coupled antibodies as isotype control were used. The representative dots present in the upper right quadrant represent the CD14+ve monocytes bind to biotinylated FAg. (**B**) Human PBMCs were incubated with biotinylated FAg with and without unlabeled FAg followed by staining with Streptavidin-FITC and analysed by FACS. Streptavidin-FITC was used as conjugate control. Representative overlaid histograms shows binding of labeled FAg are inhibited by unlabeled FAg. (**C,D**) Binding of labelled FAg of *S. digitata* and *B. pahangi* to human monocytes and murine bone marrow macrophages. Human PBMCs and murine (BALB/c) bone marrow macrophages were incubated with biotinylated FAg of *S.digitata* or *B.pahangi* or *C.elegans* for 30 minutes at 4°C followed by staining with Streptavidin-FITC and analysed by FACS. Binding to monocyte as well as lymphocyte gated populations is shown. Binding of FAg (S.digitata ; N = 34) and FAg (*B.pahangi*, *C.elegans*- N = 5) are shown for human monocytes whereas N = 10 for FAg of *S.digitata* and *B.pahangi* and N = 5 for FAg of C.elegans are shown for murine bone marrow macrophages. (**E, F**) Human PBMCs and Mouse bone marrow cells were incubated with biotinylated FAg or biotinylated AgW at 4°C for 30 minutes and stained with Streptavidin-PE and analyzed by FACS. Representative overlaid histograms shows binding of FAg, AgW to human CD 14+ve cells (**E**) and murine bone marrow cells (**F**).

**Figure 2:**
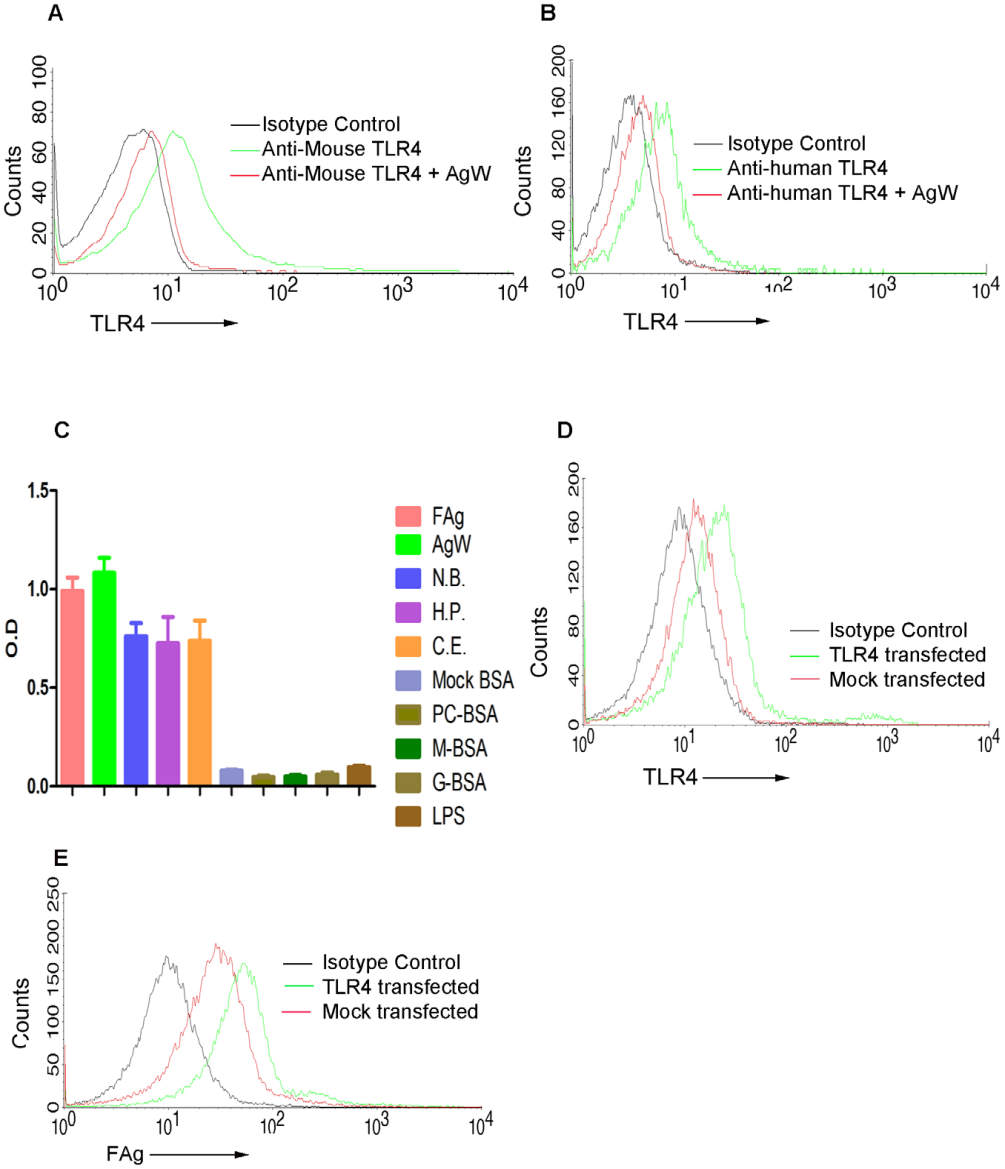
Binding OF FAg/AgW to human monocytes (in PBMCs) or murine macrophages (in BMDM) is mediated through TLR4. (**A,B**) Bone marrow cells of BALB/c mice (**A**) or purified human PBMCs (**B**) were incubated with anti-mouse TLR4-PE and anti-human TLR4-PE respectively in the presence or absence of AgW at 4°C for 30 minutes, and CD 14^+^ cells analyzed by FACS. Representative overlaid histogram shows competitive inhibition of binding of anti-mouse antibodies (**A**) or anti-human antibodies (**B**) to TLR4. (**C**) Soluble TLR4 directly binds to the helminthic antigens. ELISA plates were coated with solubilised extracts of *S.digitata*, AgW, *N.brasiliensis*, *H.polygyrus*, *C.elegans* and mock-BSA, Phosphorylcholine-BSA, GlcNAc-BSA, Mannose-BSA and LPS. After blocking with skimmed milk-PBS, human PBMC lysates were incubated. The plates were washed and incubated with rabbit anti-human TLR4 antibodies and their binding was detected by using peroxidase conjugated anti-rabbit IgG. The enzyme activity was measured using OPD. % mean ± SEM of two individual experiments are shown. (**D**) Jurkat cells trasfected with mock or TLR4 plasmid were incubated with anti-human TLR4-PE and analysed by FACS. Representative overlaid histogram shows enhanced expression of TLR4 by transfected cells as compared to mock transfected cells. (**E**) Enhanced binding to FAg after over expression of TLR 4. Mock or TLR4 transfected jurkat cells were incubated with biotinylated FAg at 4°C for 30 minutes and stained with Streptavidin-PE conjugate. The cells were thoroughly washed to remove unbound Streptavidin-PE and analysed by FACS. Representative overlaid histogram shows enhanced binding of FAg to transfected cells as compared to mock transfected cells.

Our initial studies were designed to identify an innate receptor on murine or human phagocytes that recognize nematodes. Biotinylated somatic extracts of adult stage parasites of *Setaria digitata* (FAg) and *Brugia pahangi* bound to surface of human monocytes as well as to murine bone marrow macrophages significantly more than the lymphocytes (Figure 1 **A–D**). Specificity of biotinylated FAg reacting to monocytes was confirmed by competitive inhibition with unlabeled FAg (Figure 1 B). *C.elegans*, a non-pathogenic nematode also contained a component binding to human monocytes and murine macrophages (Figure 1**C,D**). A glycoprotein (AgW) affinity purified using WGA-Sepharose column (Figure S1 **A**) was found to be the active component (Figure 1**E,F**). Since another filarial glycoprotein ES-62 has been previously reported to interact with macrophage surface through TLR4 [24], we tested such a possibility in our system. AgW as well as FAg competitively inhibited reactivity of antibodies to TLR4 on surface of murine bone marrow macrophages (Figure 2**A**, Figure S 1 **B,C**, Figure S 2 **A,B,C**) and on human monocytes (Figure 2**B**, Figure S 1 **D,E**, Figure S 2 **D,E,F**). We sought direct proof by performing a novel solid phase immunoassay developed by us. Soluble TLR4 present in membrane lysates of human PBMCs (Figure 2**C**) and murine bone marrow cells (Figure S 1 **F**) reacted with FAg/AgW bound to solid phase. Human and murine TLR4 reacted with extracts of other nematodes also viz; *Nippostrongylus brasiliensis*, *Heligomosomoides polygyrus* and *Caenorhabditis elegans* (Figure 2**C**, Figure S 1 **F**) suggesting the presence of conserved TLR4 binding components in nematodes. Enhanced binding of labelled FAg to jurkat cells over expressing TLR4 further confirmed its ability to interact with TLR4 (Figure 2**D,E**).

### Binding of FAg/AgW to TLR4 is mediated by carbohydrate moieties

**Figure 3:**
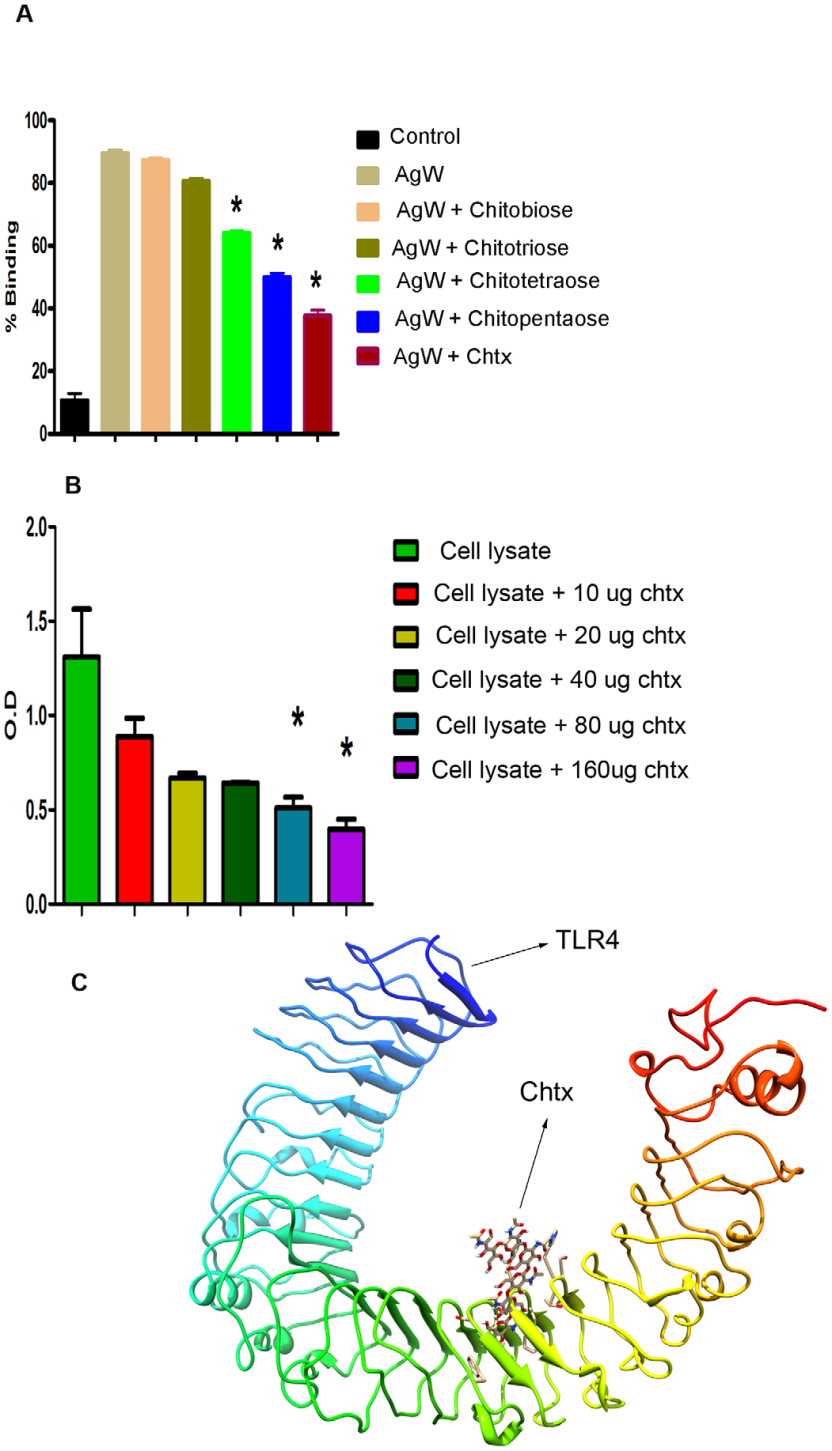
The binding of FAg to TLR4 is mediated by carbohydrate residues. Binding of AgW to human peripheral monocytes is inhibited by penta and hexa Chitooligosaccharides. (**A**) Human PBMCs were incubated with biotinylated AgW in the presence or absence of chitosugars viz; chitobiose, chitotriose, chitotetrose, chitopentaose and chtitohexaose followed by staining with Streptavidin FITC and CD14^+^ cells were analysed by FACS. % mean ± SEM of six experiments are shown. * P<0.001, versus AgW binding without chitosugars (Student's t-test). (**B**) Binding of AgW to TLR4 is inhibited by chtx. ELISA plates were coated with AgW. After blocking with skimmed milk-PBS, human PBMC lysate were pre incubated with different concentrations of chtx for 30 minutes at 37°C and were then transferred to AgW coated plates. Cell lysates without pre incubation with chtx was used as control. The plates were thoroughly washed and were incubated with rabbit anti-human TLR4 and its binding was detected by using peroxidase conjugated anti-rabbit IgG. The enzyme activity was measured using OPD as substrate. % mean ± SEM of two experiments are shown * P<0.001, versus cell lysates without preincubated with chtx (Student's t-test). (**C**) In silico analysis between chtx and TLR4. Three dimensional molecular docking between chtx and extra cellular domain of mouse TLR4 was performed using patch dock software demonstrating the interaction of chtx to the internal pocket of extra cellular domain of TLR4.

Since immunomodulatory properties of helminth products have been shown to depend on glycan moiety [25] we examined the possible role of carbohydrates in FAg-TLR4 interaction described above. Deglycosylated (Figure S 3 **A**) or chitinase treated (Figure S 3 **B**) FAg failed to competitively inhibit interaction of labeled FAg with monocyte surface suggesting the involvement of carbohydrate residues in such interactions. Susceptibility to chitinase also indicated the role played by chitin or it's oligomers in FAg interacting with TLR4. This was tested by competitive inhibition using chitin oligomers. Binding of AgW to human monocytes was inhibited by chitosugars of varying size, longer the chain length higher was the degree of inhibition (Figure 3**A**). Further, the hexasaccharide, chitohexaose (chtx) also inhibited reactivity of soluble TLR4 to AgW on solid phase in a dose dependant manner (Figure 3**B**). These results suggested involvement of chitosugar residues present in FAg/AgW in interaction with TLR4. *In silico* analysis using crystal structure of TLR4 and 3D structure of chtx (http://pubchem.ncbi.nlm.nih.gov/summary/summary.cgi?cid=197182&loc=ec_rcs) also indicated significant affinity between the two molecules (Figure 3**C**, Figure S 3 **C**). Based on these observations further biological characterization were carried out using chtx as described below.

### Chtx inhibits LPS induced production of inflammatory mediators *in vitro*

**Figure 4:**
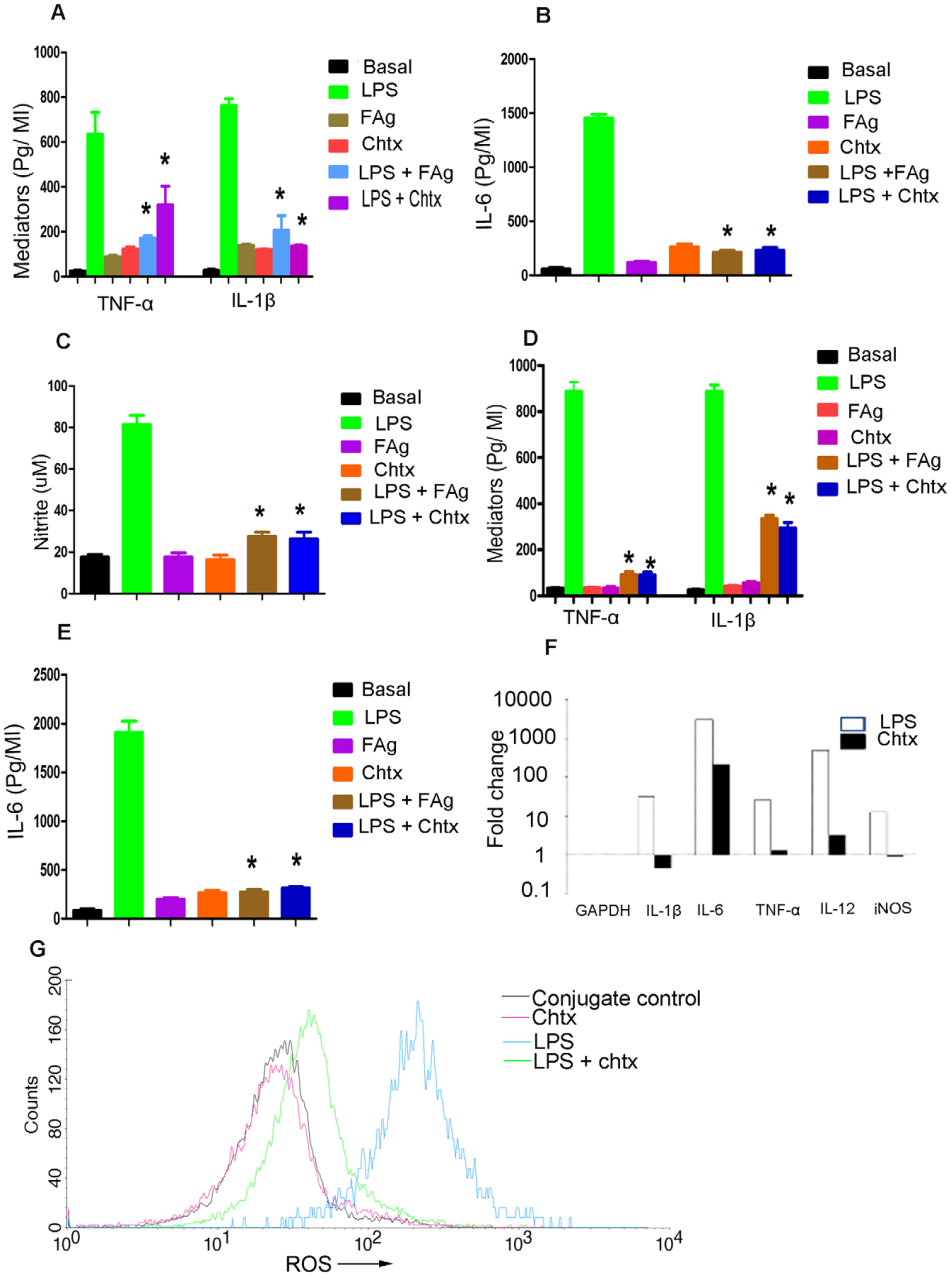
Chtx or FAg inhibits LPS induced inflammation. Human monocytes and BMDM of BALB/c mice were stimulated with FAg, chtx with and without LPS for 48 hrs. TNF-α, IL-1β, Il-6 and nitrite in culture supernatants were quantified according to the manufacturer's instruction. TNF-α, IL-1β, IL-6 and nitrite in culture supernatants for mice (Fig. 4**A,B,C**) and TNF-α, IL-1β and IL-6 in human (Fig. 4 **D,E**) are shown * P<0.001, versus LPS treated cells (Student's t-test). N = 21 for human control, LPS, FAg, LPS+FAg and for chtx and chtx+LPS N = 10. N = 15 for mouse control, LPS, FAg, LPS+FAg and for chtx and chtx +LPS N = 10. (**F**) LPS induces significant increase of proinflammatory mediators in comparison to chtx as shown by RT-qPCR. Bone marrow derived macrophages were treated with LPS and chtx for 6 hrs. The cells were harvested and total RNA were isolated from the cells. Expression of TNF-α, IL-1β, IL-6 and iNOS genes was analysed. (**G**) Cultured BMDM of BALB/c mice were pre incubated with 5 µM H_2_-DCFDA before treatment with LPS, chtx or mixture of both. The cells were harvested after 24 hrs, washed with PBS twice and analysed by FACS. Representative histogram shows generation of ROS by LPS and their inhibition by chtx.

The above findings that chtx residues present in FAg or AgW binds to TLR4 opened up the possibility of using the small molecular weight chito-oligosaccharide as a potential TLR4 antagonist to block LPS mediated inflammatory responses. BMDM of normal mice and normal human PBMCs were stimulated with LPS or chtx for 48 hrs and levels of TNF-α, IL-1β, IL-6 and nitrites were quantified in culture supernatants. LPS stimulated significant production of inflammatory mediators while chtx failed to do so in both murine and human systems (Figure 4 **A–E**). Induction of genes for production of inflammatory molecules by LPS and failure to up regulate such genes by chtx was confirmed by Q-PCR also (Figure 4**F**). Chtx on the other hand significantly inhibited LPS mediated inflammatory activation of mononuclear cells in both the systems (Figure 4 **A–E**). We then tested induction of reactive oxygen species (ROS) by LPS and chtx. Murine BMDMs upon stimulation with LPS significantly up regulated ROS while chtx failed to do so. Rather chtx significantly inhibited LPS induced up-regulation of ROS. (Figure 4**G**) This observation is significant in the context of a recent report suggesting that induction of inflammatory cytokines is dependent on up regulation of ROS [26].

### Chtx inhibits production of inflammatory mediators and protects mice from endotoxemia

**Figure 5:**
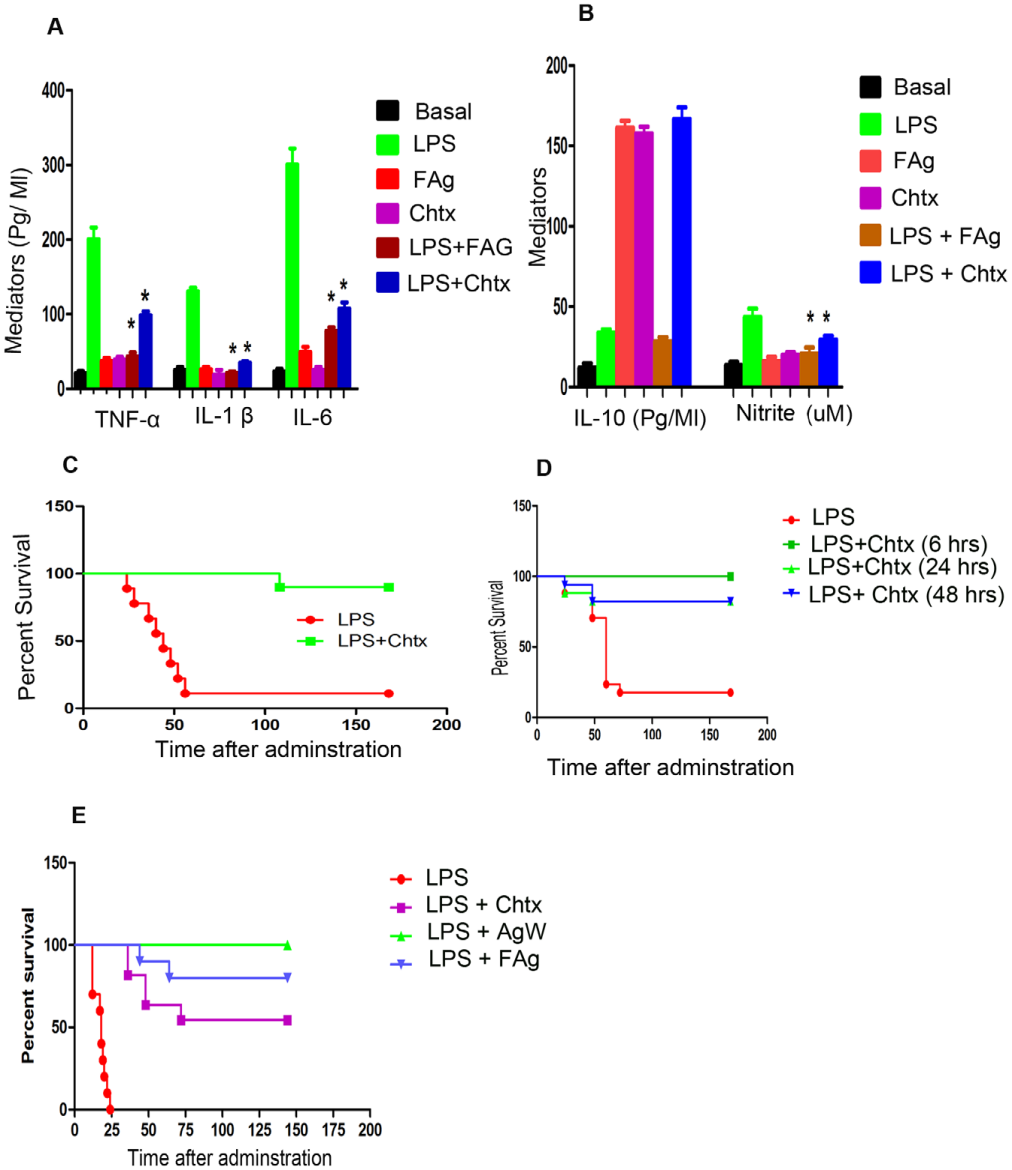
Chtx protects mice from endotoxic shock. Chtx and FAg inhibit induction of pro inflammatory cytokines in vivo by LPS. Eight to ten weeks old BALB/c mice were injected IP with 15 mg/Kg body wt of LPS with or without FAg (100 µg) and chtx 250 µg and blood was collected by heart puncture in heparinised tubes after 2 hrs. Plasma levels of TNF-α, IL-1 β and IL- 6 (Fig. 5 **A**) and IL-10 and nitrite (Fig. 5 **B**) were quantified as described earlier. Mean ± SEM of 10 independent experiments are shown. * P<0.001, versus LPS treated cells (Student's t-test). (**C**) C57 BL/6 mice were injected IP with lethal dose of LPS (60 mg/Kg body wt) with or without chtx (250 ug) and mortality was scored. (N = 15). (**D**) C57 BL/6 mice were injected IP with lethal dose of LPS (60 mg/Kg body wt) and chtx was injected later as shown and mortality scored. N = 17 for LPS, N = 10 for LPS+Chtx 6 hrs, N = 16 for LPS+Chtx 24 hrs and LPS+chtx 48 hrs. (**E**) BALB/c mice were injected IP with lethal dose of LPS (15 mg/Kg body wt.) with or without FAg (100 µg) or AgW (50 µg) or Chtx (250 µg) and the mortality was scored. N = 10.

For *in vivo* validation of the above observations, BALB/c mice were administered a lethal dose of LPS with and without chtx. Chtx significantly inhibited LPS induced increase in plasma levels of TNF-α, IL-1β, IL-6 and nitrites (Figure 5 **A,B**) while increasing IL-10 levels (Figure 5**B**). More critically, chtx significantly blocked LPS induced mortality of C57BL/6 mice (Figure 5**C**) suggesting that it can be used as a potential antagonist to block adverse biological consequences of endotoxemia *in vivo*. Administration of chtx 6, 24 and 48 hrs after onset of endotoxemia was also effective in blocking mortality of mice suggesting its potential as a therapeutic agent (Figure 5 **D**). BALB/c mice which are more susceptible to LPS induced endotoxemia than C57BL/6 mice were also protected by chtx (Figure 5**E**).

### Chtx induces alternate activation of monocytes/macrophages *in vitro*

**Figure 6:**
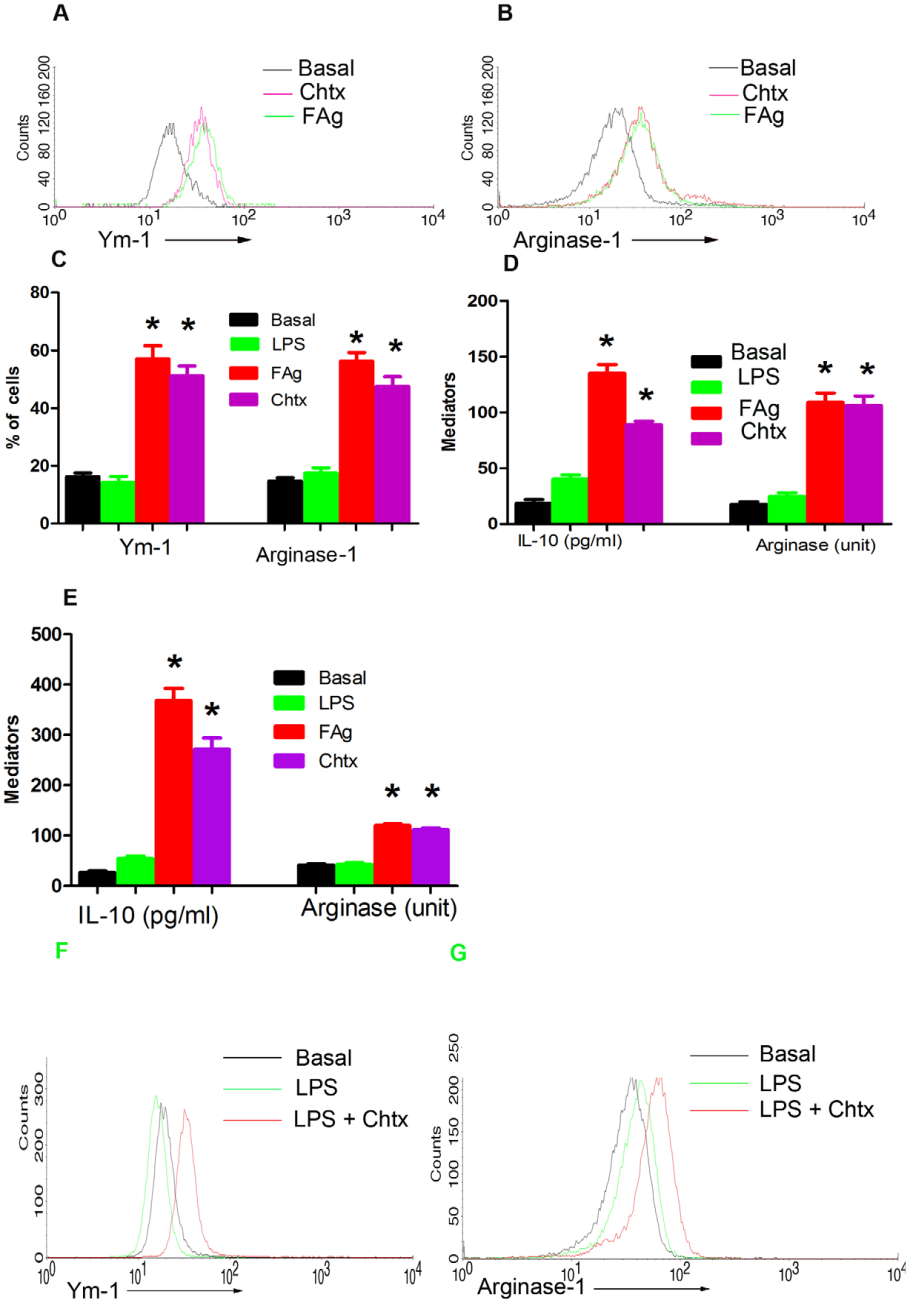
FAg or chtx induce alternate activation of both human monocytes and murine macrophages. Human monocytes and BMDM of BALB/c mice were stimulated with FAg, chtx or LPS for 48 hrs. IL-10 in culture supernatants were quantified according to the manufacturer's instruction. Both human and mice cells were analysed for Arginase activity and additionally mice cells were analysed for Ym-1 and Arginase-1 by intracellular staining. (**A,B,C**) Expression of Ym-1 and Arginase-1 in mouse BMDM upon stimulation with FAg and chtx (N = 10). (**D**) Induction of IL-10 and up-regulation of Arginase activity in BMDM upon stimulation with FAg and Chtx (N = 10). (**E**) Induction of IL-10 (N = 21 for basal, LPS, FAg and 10 for chtx) and up-regulation of Arginase activity in human PBMCs upon stimulation with FAg and chtx (n = 10). LPS failed to induce IL-10, Arginase-1 and Ym-1 (**C,D,E**). * P<0.001, versus LPS treated cells (Student's t-test). (F, G) Expression of Ym-1 and Arginase-1 by peritoneal macrophages analyzed *ex-vivo* after administration of LPS at time 0 and chtx at time 6 hrs. Peritoneal macrophages collected 90 minutes post chtx administration were stained for Ym-1 or Arginase-1 were analysed.

Alternate activation of macrophages has been well documented in helminth infections [27]. Presence of abundant chitin in helminthes [28] and activation of chitinase family proteins like AMCase, Ym-1 and chitinase-3 during helminth infections [29] suggests that chitin breakdown products from helminthes could be responsible for alternate activation of macrophages. We explored this possibility by stimulating human PBMCs and murine BMDM with chtx. Ym-1, Arginase-1 and IL-10 (Figure 6 **A–D**, Figure S 4) were up-regulated by murine BMDM upon stimulation with chtx. Similarly, human monocytes released IL-10 (Figure 6 **E**) and up-regulated intracellular Arginase activity confirming induction of alternate activation of mononuclear cells by chtx *in vitro* (Figure 6 **D,E**).

### Chtx induces alternate activation of macrophages *in vivo*

The potential of chtx to induce alternate activation of macrophages *in vivo* was also addressed. BALB/c mice were intraperitoneally administered with chtx or LPS and after 90 minutes the peritoneal cells were harvested and expression of Ym-1 and Arginase-1 in CD14+ve cells were analyzed. Chtx up regulate both Ym-1 and Arginase-1 while such an up-regulation was not observed in LPS administered mice peritoneal macrophages (Figure S 4 **G,H**). Further, *in vivo* alternate activation of macrophages by chtx even after onset of endotoxemia was demonstrated. Up-regulation of Ym-1 and Arginase-1 was observed in peritoneal macrophages collected 90 minutes post chtx administration in mice with ongoing endotoxemia. It was observed that chtx induce alternate activation of macrophages even after onset of endotoxemia (Figure 6 **F,G**).

### Alternate activation of macrophages induced by chtx is mediated by TLR4

**Figure 7:**
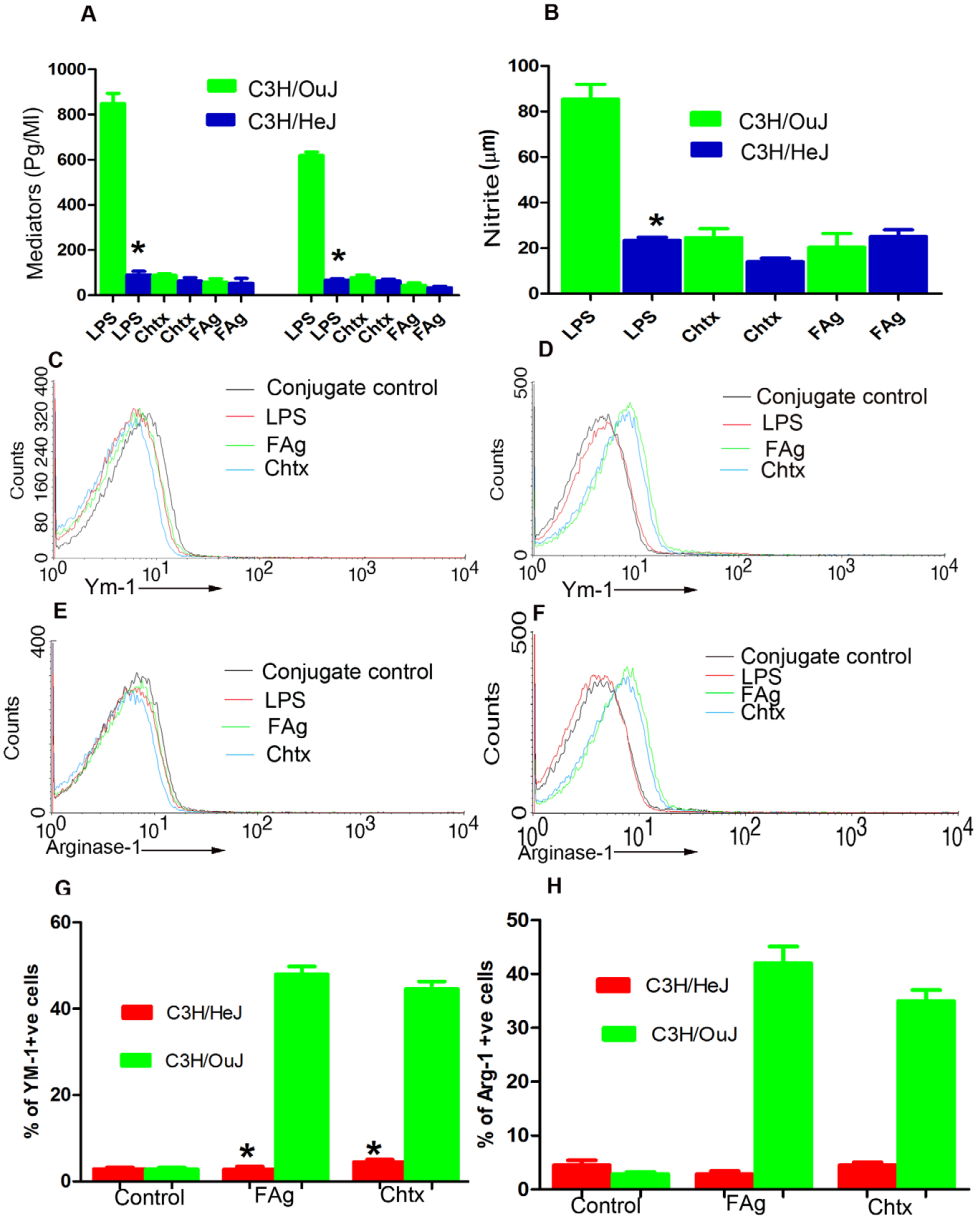
Alternate activation of macrophages induced by chtx is mediated by TLR4. Bone marrow cells of C3H/HeJ and C3H/OuJ were stimulated with LPS, chtx or FAg for 48 hrs. (**A,B**)TNF-α, IL-1 β and nitrite levels in culture supernatants were quantified. % mean ± SEM of three experiments are shown * P<0.001, versus C3H/OuJ cells (Student's t-test). (**C–F**) Role of TLR4 in activation of macrophages differentially by LPS and chtx. BMDM of C3H/HeJ and C3H/OuJ mice were stimulated with FAg, LPS and chtx for 48 hrs. Intracellular expression of YM-1 and Arginase-1 was scored. Representative overlaid histogram shows the up regulation of Ym-1 and Arginase-1 upon stimulation with FAg, chtx only in wild type C3H/OuJ mice (**D,F**) and not inC3H/HeJ (**C,E**) whereas LPS fail to up-regulate Ym-1 and Arginase-1 expression in both the strain of mice. % mean ± SEM of five individual experiments for Ym-1 (**G**) and Arginase-1 (**H**) are shown.

Chitin and chitin breakdown fragments have been reported extensively to activate macrophages but the pathway of activation and receptors involved remain contradictory [30]. The above described results suggested that chtx activates macrophages through alternate pathway using TLR4. We sought direct biological proof by stimulating BMDMs of C3H/HeJ (TLR4 mutant mice) [31] and C3H/OuJ (wild type mice) with chtx and LPS and canonical markers of both alternate as well as classical macrophage activation were scored. As expected, LPS induced release of inflammatory cytokines by BMDMs of wild type mice and not by cells of mutant C3H/HeJ mice (Figure 7 **A,B**). Chtx on the other hand failed to induce classical activation markers viz; TNF-α, IL-1β and nitrite (Figure 7 **A,B**) but up-regulated expression of alternate activation markers viz; Ym-1 (Figure 7 **C,E**) and Arginase-1 (Figure 7 **D,F**) in wild type mice and not in mutant mice. From these observations we make two broad conclusions: a) classical and alternate macrophage activation could be mediated using the same receptor, TLR4 by LPS and chtx respectively and b) the mutation in TLR4 gene that results in substitution of proline to histidine in its intracellular domain [32] plays a critical role in both classical as well as alternate activation pathways in macrophages.

### Monocytes in filariasis infected subjects display alternate activation phenotype and are LPS resistant

**Figure 8:**
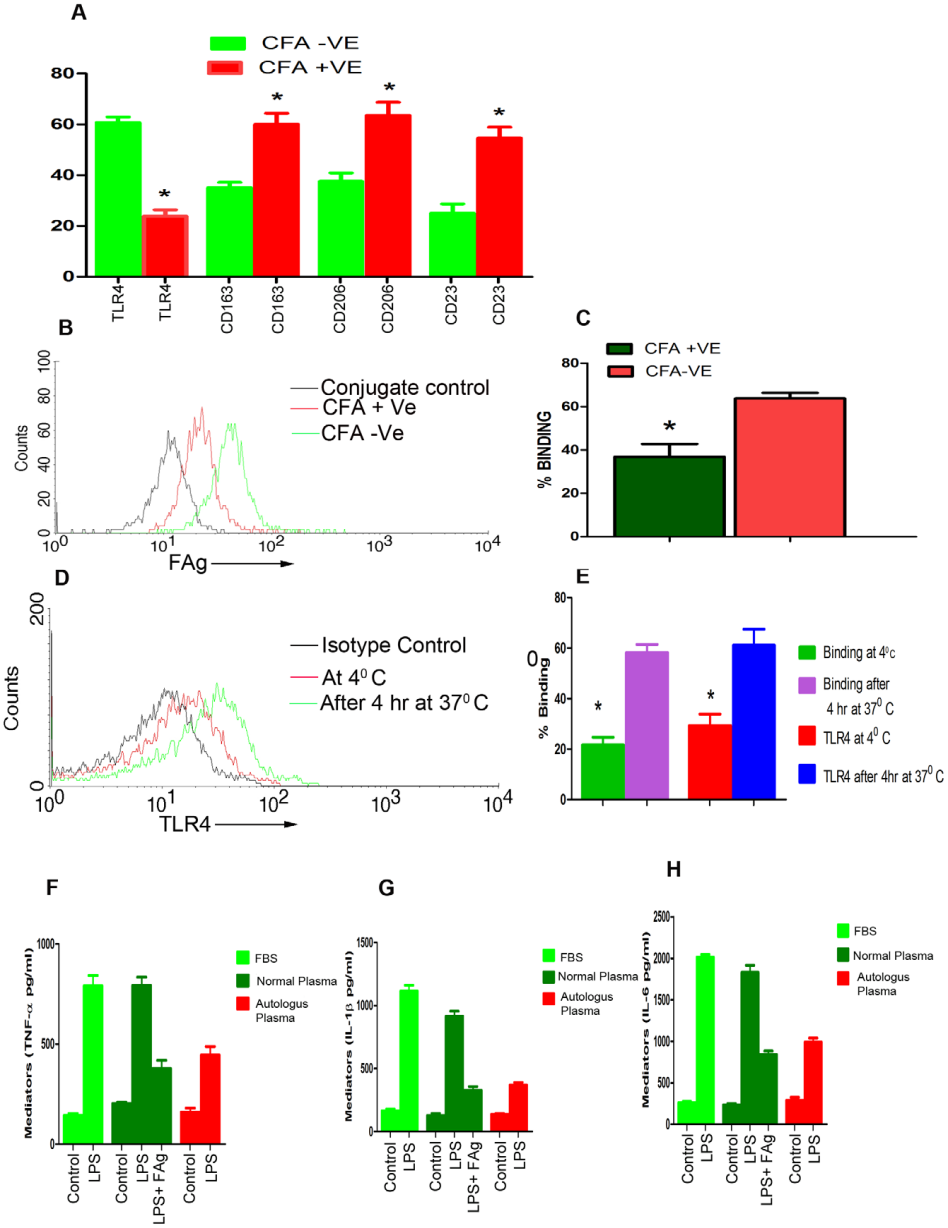
Monocytes of subjects with active filarial infection are alternately activated and less responsive to LPS induced signaling. (**A**) PBMCs (suspended in medium containing autologous plasma) of subjects with active filarial infection and endemic controls were stained for TLR4 (N = 10), CD23 (N = 8), CD163 (N = 11) and CD206 (N = 10) along with CD14. Cells gated for CD14 were analysed by FACS. * P<0.001, versus CFA (Circulating Filarial Antigen)−ve cells (Student's t-test). (**B**) PBMCs (suspended in medium without autologous plasma) of subjects with active filarial infection and endemic controls were incubated with biotinylated FAg at 4°C for 30 minutes followed by staining with Streptavidin-FITC and analysed by FACS. Representative overlaid histogram shows binding of FAg to monocytes of subjects with active filarial infection and endemic control. (**C**) Same as 8B but performed in 12 subjects with active filarial infection and 34 endemic controls FAg. * p<0.001 versus endemic controls. (**D**) PBMCs of subjects with filarial infection (CFA+ve) were incubated at 37°C for 4 hrs and then incubated with anti-human TLR4-PE and analysed by FACS. PBMCs incubated at 4°C and stained with anti-TLR4 were taken for comparison. Representative overlaid histograms are shown for CD14+ve monocytes. (**E**) PBMCs of 5 human subjects with filarial infection (CFA+ve) were incubated at 37°C for 4 hrs and then incubated with anti-human TLR4-PE or biotinylated FAg at 4°C for 30 minutes followed by staining with streptavidin-FITC and CD14 gated population were analysed by FACS. PBMCs incubated at 4°C were taken for comparison. (**F,G,H**) PBMCs of 10 human subjects with active filarial infection were cultured in medium containing 10% autologus/heterologous normal plasma or 10% FBS in humidified atmospheric condition (5% CO_2_, 80% humidity). After 8–10 hrs incubation at 37°C non adherent cells were removed by washing with sterile medium. The remaining adherent cells were stimulated with LPS or LPS along with FAg for 48 hrs. The culture supernatants were analyzed for TNF-α (Fig. 8 **F**) and IL-1β (Fig. 8**G**) and IL-6 (Fig. 8**H**).

Finally we addressed the significance of these observations in human lymphatic filariasis. The expression of TLR4 was significantly low on monocytes of infected subjects positive for CFA (Circulating Filarial Antigen) in comparison to endemic controls (negative for CFA ) (Figure 8**A**). Binding of labeled FAg to monocytes was also significantly less in infected subjects in comparison to endemic controls (Figure 8**B,C**) however when monocytes of infected subjects were incubated in vitro at 37°C for 4 hrs binding of labeled FAg as well as expression of TLR4 were comparable to endemic controls (Figure 8**D,E**). We interpret these findings to imply that circulating filarial antigens saturate TLR4 on monocytes which get recycled when incubated in culture thus exposing surface TLR4 to bind FAg or to react with anti-TLR4 (Figure 8**E**). Further, LPS induced inflammatory molecules by PBMCs of infected subjects was significantly decreased in comparison to controls as shown by TNF-α, IL-1β and IL-6 (Figure S 5 **A–C**) levels in culture supernatants suggesting that monocytes of subjects with filarial infections are less prone for activation by LPS possibly due to saturation of TLR4 with circulating filarial antigens. This issue was further addressed by incubating monocytes of infected individuals with heterologous (negative for CFA) normal plasma and FBS and stimulated with LPS or with LPS+FAg and inflammatory molecules like TNF-α, IL-1β and IL-6 were quantified in culture supernatants. LPS induced inflammatory cytokines by monocytes of infected individuals are comparable with monocytes of endemic controls when incubated with normal heterologous plasma or with FBS suggesting that filarial antigen in infected plasma saturate TLR4 on monocytes thus blocking activation by LPS (Figure 8 **F,G,H**). Expression of CD23, CD163, and CD206 canonical markers of alternate macrophage activation [33] were significantly up-regulated on circulating monocytes of infected subjects in comparison to uninfected controls (Figure 8 **A**).

## Discussion

Three novel issues stand out from the results being reported in this communication-a) that a small molecular weight carbohydrate, chtx activates macrophages to a non-inflammatory phenotype through TLR4 and in doing so functions as an LPS antagonist and blocks induction of inflammatory mediators by LPS *in vitro* (both in murine macrophages and in human monocytes) and endotoxemia *in vivo*, b) that two diverse pathways of activation of macrophages could be operational by two different ligands viz; LPS and chtx using a single receptor TLR4 on the host cell and finally c) that glycoproteins of filarial nematodes with chtx as a constituent could be saturating TLR4 on circulating monocytes in infected subjects rendering them refractory to LPS induced inflammatory activation.

We have provided evidence for direct binding of AgW to TLR4 by flow cytometry and consequent activation of alternate pathway of monocytes/macrophages. We have also shown by in silico analysis and solid phase immunoassay that chtx also binds toTLR4 and that the phenotype of activation pathway by chtx is similar to that of AgW. It is critical to note that unlike AgW, native LPS does not bind to TLR4 - several investigators have provided convincing evidence to suggest that native LPS does not bind directly to TLR4 but it activates macrophages by forming a complex with LPS binding protein (LBP) and CD14 and this complex delivers LPS to MD2 which activates macrophages through TLR4 [9]–[11]. When viewed in this context our findings on direct binding of AgW to TLR4 and activation of macrophages by alternate pathway is fundamentally different from macrophage activation by LPS which does so without directly binding to TLR4 in its native form.

Benefits of inhibition of TLR4 activation has been documented in several experimental models of lethal shock. Anti-CD14 antibodies in rabbits, primates and humans [34]–[36], anti-TLR2 antibodies [37] and antibodies to TLR4 [15] or TLR4/MD2 complex [17] have been tested with a high degree of success. Synthetic LPS antagonists such as Eritoran and Tak-242 have been tested in experimental models of endotoxic shock and also in human disease [19] but Eritoran has been recently reported to be ineffective in phase-III clinical trial (http://clinicaltrials.pharmaceutical-business-review.com/news/eisai-eritoran-fails-to-meet-primary-endpoint-in-phase-iii-trial-250111). Molecules involved in downstream signaling of TLR such as platelet-activating factor [38] oxidized phospholipids [22], [23], nitrate salts [20], 5c [21] have also been tested with a degree of success. Potential of antibodies to TNF-α, IL-1RA, TNF-α soluble receptors and anti-bradykinin have also tested [39] but it has been observed that treatment with such TLR inhibitors interfere with innate immunity of host against infection and consequently increasing the risk of shock and mortality [39], [40], [41]. In this context the results of this study offers significant promise- a non immunogenic inexpensive small molecular weight chito-oligosaccharide can be used as an LPS antagonist. Based on *in vitro* demonstration of alternate activation of murine macrophages and human monocytes and *in vivo* activation of murine macrophages into alternate phenotype by chtx leading to inhibition of LPS mediated induction of inflammatory molecules (such as IL1β, TNF-α, IL-6 etc) by chtx we conclude that the small molecular weight carbohydrate induces alternate activation of macrophages *in vivo* and mediates protection against endotoxemia. Thus Chtx appears to protect against endotoxemia by two mechanisms - a) it competitively inhibits LPS induced activation by binding to TLR4 and/or b) it activates macrophages by alternate anti-inflammatory pathway. Generation of such an activation appears to have many advantages since alternately activated macrophages are reported to be endotoxin resistant [42], [43] with increased phagocytic activity [44] and enhanced expression of scavenger receptors and proangiogenic factors [45] make them assist in tissue repair and resolution of inflammation [46]. We are currently testing if monocytes of sepsis patients can be re-programmed to non-inflammatory state by chtx.

LNFP-III a complex carbohydrate moiety of *S.mansoni*, thioredoxin peroxidase of *F.hepatica* and migration inhibition factor (MIF) of *B.malayi* [47], [48] are other molecules of helminth origin reported to induce alternate activation of macrophages but the host receptor through which such activation is mediated is still largely unknown. The current study demonstrating the role of TLR4 in chtx induced alternate activation of macrophages has offered insights into the issue of induction of alternate activation by helminth products. Although there have been many studies examining TLR signaling in response to pathogens [49]–[51] fewer studies have examined interaction of multicellular helminth parasites with TLRs on monocytes or macrophages. Helminth products such as excretory secretory (ES) product of *Necator americana* or OV-Asp-1 of *Onchocerca volvulus* [52], [53] are known to interact with cells of the innate immune system but the receptor associated with this interaction has not been elucidated. In the present study a filarial glycoprotein designated as AgW has been demonstrated to bind directly to TLR4. These results are in broad agreement with demonstration of ES-62, a filarial glycoprotein interacting with TLR4 [24]. Apart from identifying TLR4 as a receptor for a helminth carbohydrate, the current study will be of crucial interest to cell biologists since TLR4 appears to function as a common receptor for both classical as well as alternate activation of macrophages and the nature of ligand determining the phenotype. This clearly offers scope for acquiring insights into molecular events involved in mutually exclusive activation pathways of macrophages.

Human filariasis is characterized by chronic persistence of circulating filarial antigens (CFA) for several years. TLR4 on monocytes in infected subjects appear to be saturated *in vivo* with CFA since labeled FAg bound poorly bound to monocytes *ex vivo* and normal binding of FAg to TLR4 could be achieved by allowing antigen saturated TLR4s to recycle *in vitro*. The findings reported here suggest that CFA in plasma seem to remain bound to TLR4 on monocyte surface in infected subjects and contribute to sustenance of their alternate activated state. The following observations indicate that inherent defect in monocytes of infected subjects do not contribute to their failure to get activated by LPS: 1. Monocytes of infected subjects when incubated with infected (autologous) plasma, the response to LPS was significantly diminished (as shown by decreased TNF-α, IL-1β and IL-6 levels in culture supernatants) when compared with response of same monocytes incubated with FBS or normal plasma; 2. Monocytes of infected subjects cultured with normal plasma respond poorly to LPS when FAg was added exogenously; 3. FAg significantly inhibited release of TNF-α, IL-1β and IL-6 by normal monocytes when cultured with autologous plasma and stimulated with LPS; 4. When monocytes of infected subjects are incubated with FBS or with normal human plasma, the cells get activated well to LPS as shown by higher levels of inflammatory cytokines in supernatants - TNF-α, IL-1β and IL-6 levels are comparable to those observed in normal monocyte cultured with normal (autologous) plasma . While our observations that circulating monocytes in filariasis infected subjects display alternate activation markers are similar to an earlier report [54] diminished induction of mediators of inflammation such as TNF-α, IL-1β and IL-6 by LPS treated monocytes of filariasis infected subjects is a novel observation. The possibility that subjects with filarial infections will be regulating hyper inflammation associated with bacterial infections and thus offering protection against endotoxemia associated with sepsis needs further investigation. These findings also suggest interesting evolutionary issues on co-infection of humans with nematodes and gram negative bacteria and their pathogenesis. It is tempting to propose that increasing incidence of sepsis/septic shock in developed countries over the last 100 years (2) could have been due to eradication of helminth infections, a scenario similar to increased incidence of allergies and autoimmune diseases in developed countries as a consequence of elimination of infectious disease as proposed in ‘Hygiene Hypothesis’.

## Materials and Methods

### Ethics statement

Institutional Animal Ethics Committee of Institute of Life Sciences “approved” all the protocols followed for experiments conducted using mice. The study was carried out in strict accordance with the recommendations of the Committee for Prevention of Cruelty and safety of experiments with animals (CPCSEA) a regulatory body of Government of India that supervises Care and Use of Laboratory experimentation through their nominees in the Institutional animal ethics committee. Adult *Setaria digitata* worms from peritoneal cavities were collected from the abattoir attached to a local zoo after obtaining approval from zoo authorities. The animals are slaughtered in the abattoir regularly for feeding wild cats and no animals were slaughtered specifically for the purpose of our study. The study on human filariasis was approved by Institutional Human Ethics Committee of Institute of Life Sciences which operates under the guidance of regulations of Indian Council of Medical Research. Written informed consents were obtained from each of the normal control volunteers, filariasis infected persons and/or their legal guardians before collection of blood samples.

### Collection of bovine adult filarial parasites (*S.digitata*)

Peritoneal dwelling adult female filarial parasites (*Setaria digitata*) were collected from cattle in a local abattoir, attached to the local zoological park at Nandankanan, Bhubaneswar after obtaining necessary approval from zoo authorities. The worms were transported to the laboratory in Dolbecco's Modified Eagles Medium (DMEM) (Sigma) pH 7.00 containing antibiotics [Penicillin Streptomycin solution 1 ml/100 ml of medium] (Sigma) and 1% glucose (Hi-media).

### Preparation of filarial extracts and labeling

Aqueous extracts of *S. digitata* (designated as FAg) was prepared by homogenization followed by ultrasonication and the aqueous extract of adult worms was biotinylated using appropriate derivatives of biotin i.e. N-hydroxysuccinamide derivatives (Sigma) suitable for protein labeling.

### Preparation of AgW

One milligram of FAg was passed through WGA-Sepharose (Sigma) coloumn and the unbound proteins were washed by passing PBS and glycoproteins bound to WGA were eluted by Glycine-HCl buffer (pH 3.6).The pH of the elutes was adjusted using 0.1 M NaOH and dialysed against PBS. Protein concentration of the elute was estimated and stored at −20°C for further use.

#### LAL Assay

Batches of purified AgW and FAg were tested by LAL assay for absence of endotoxin as per the manufactures instruction using commercially available LAL assay kit (MP Biomedicals) and endotoxin free water was used for diluting both AgW and chtx before use in cell culture.

### Animals

BALB/c, C57BL/6, C3H/OuJ and C3H/HeJ mice were obtained from National Institute of Immunology, New Delhi which was originally imported from Jackson laboratories, Germany. Breeding and maintenance were done at the animal facility at Institute of Life Sciences, Bhubaneswar, India. 8–10 weeks old animals were used for this study. Institutional animal ethics committee of Institute of Life Sciences, Bhubaneswar approval was obtained for all the investigations conducted in mice.

### Bone marrow cells culture

Mouse bone marrow cells were collected from femoral shafts by flushing with 3 ml. of cold sterile DMEM (Sigma) supplemented with 20 mM of L-Glutamine (ICN), antibiotics (1 ml penicillin and streptomycin/100 ml of medium) (Sigma)containing 10% FBS. The cell suspension was passed through a sieve to remove large clumps. The cell suspension was washed 2–3 times with sterile DMEM and adjusted to 0.5×10^6^ cells/well and cultured in 24 well plates. After 8–10 hrs incubation at 37°C non adherent cells were removed by washing with sterile DMEM and the adherent cells (more than 96% positive for CD14 (Figure S-4 and S-6 indicating high purity) were stimulated with LPS (Sigma, 055:B5 L-2880) with or without FAg or chtx (Dextra Lab) at 10 µg/ml concentration. After 48 hrs the supernatants were aspirated, frozen at −80°c and used later for estimation of cytokine levels. The adherent cells were removed using chilled medium and analysed for intracellular Arginase activity by calorimetric assay and intracellular staining using antibodies to Ym-1 and Arginase-1 staining and scored by flowcytometry.

### Mouse model of Endotoxemia

8–10 weeks old BALB/c and C57BL/6 mice were intraperitoneally injected with 15 mg/Kg body wt and 60 mg/Kg body wt. of LPS respectively with and without FAg (100 µg) or AgW (50 µg ) or chtx (250 µg) and observed for mortality a period over 168 hrs. These doses were determined by prior titration and the lowest concentration effective in vivo was chosen for experimentation. For analysis of levels of cytokines mice were sacrificed 2 hrs after challenge with LPS or LPS with FAg (100 µg) or LPS with chitohexaose (250 µg). Blood was collected in heparinised tubes by heart puncture and clear plasma was isolated by centrifugation at 5000 g for 10 minutes and analysed for presence of TNF-α, IL-1β, nitrite and IL-10 as described below.

#### Human blood samples

The study population was drawn from areas in and around Bhubaneswar, Orissa; highly endemic for Bancroftian filariasis. About 5 ml. of nocturnal blood each was collected in heparinised glass vials from the selected human study population as per the ethical guidelines of ILS, Bhubaneswar, Orissa. The patients were classified into different clinical categories according to the criteria described by our group earlier [55]. Subjects with active filarial infection were identified as described and blood samples were collected from subjects with circulating filarial Antigen (CFA) positive and those free of infection for this study. The study on human filariasis was approved by Institutional Ethics Committee of ILS and written consents were obtained from normal as well as infected persons before collection of blood samples.

### Isolation of human PBMCs and *in vitro* culture

Human PBMCs were isolated from heparinised venous blood samples by density gradient centrifugation method using Histopaque (Sigma). Briefly, the heparinised blood was layered on LSM medium gently in the ratio of 1∶1 and subjected to centrifugation at 100 g for 30 minutes. The white layer representing PBMCs was aspirated out gently and transferred aseptically into sterile centrifuge tubes. The suspension of cells was then washed and cultured in sterile DMEM supplemented with 20 mM of L-Glutamine (ICN), 10% of autologus plasma/FBS and antibiotics (1 ml penicillin and streptomycin/100 ml of medium) (Sigma).The no. of cells was adjusted 0.5×10^6^ cells/well in 24 well plates. After 8–10 hrs incubation at 37°C non adherent cells were removed by flushing with sterile DMEM and the adherent cells were stimulated with LPS, FAg or chtx, LPS along with FAg and LPS along with chtx at 10 µg/ml concentration for 48 hrs. after which the supernatants were removed and used for cytokine estimation. The adherent cells were removed and analyzed for intracellular Arginase activity by calorimetric assay as described below.

For study of recycling of the receptor, the PBMCs were resuspended in DMEM containing 0.1% BSA and incubated at 37°C for 4 hrs. Then cells were incubated with biotinylated FAg at 4°C for 30 minutes followed by staining with streptavidin-FITC and analyzed by FACS.

### Quantification of cytokines

Supernatants from human monocytes or mouse BMDM cultures as well as mice plasma samples were analysed for levels of TNF-α, IL-1β, IL-6 and IL-10 by a sandwich ELISA according to manufacturers instruction using commercially available ELISA kits (e-Biosciences).

### Flow cytometry

Human PBMCs or mouse BMDM (1×10^6^/ml) were stained for 30 minutes at 4°C with fluorescence labeled antibodies specific to CD14, TLR4 (e Biosciences) mixed with and without FAg or AgW along with relevant isotype controls. Human cells were also stained with antibodies to CD23, CD163, and CD206 (Santacruz Biotech.).The cells were thoroughly washed to remove the unbound antibodies and analysed by FACS (BD FACS caliber).

For intracellular Arginase-1 and YM-1 staining, cells were permeabilised with 1× FACS permeabilising solution (BD biosciences) and then incubated with rabbit antibodies to mouse YM-1 (Stemcell technologies) or goat anti-mouse Arginase-1 antibodies (Santacruz Biotech.) followed by staining with anti-rabbit IgG-FITC(Sigma) and anti-goat IgG-PE (Santacruz Biotech.) respectively and analysed by FACS. Appropriate isotype controls/conjugate controls were used for all flowcytometric assays.

### Preparation of PBMC and bone marrow cell lysates

Human PBMCs or mouse (BALB/c) bone marrow cells (2×10^6^) were incubated with 2 ml of cell lysis solution (Sigma) and a cocktail of protease inhibitors (Sigma) for one hr. at 4°C and then ultra-sonicated. The supernatant was collected by centrifuging at 1500 g for 10 minutes and stored for further use.

### Enzyme Linked Immunosorbent Assay

ELISA plates (Nunc maxisorp) were coated with 1 µg of PBS extracts of *S.digitata*, AgW, *N.brasiliensis*, *H.polygyrus* or mock BSA, Phosphorylcholine coupled to BSA, GlcNaC-BSA, Mannose-BSA or LPS. After blocking with 1% skimmed milk-PBS (Hi-media), human PBMC lysates or mouse (BALB/c) bone marrow cell lysates were incubated for 2 hr at 37°C.The plate was thoroughly washed and were incubated with rabbit anti-human and rabbit anti-mouse TLR4 (e Biosciences) respectively. The binding of anti-human and anti-mouse TLR4 was detected by using peroxidase conjugated anti-rabbit IgG (Sigma). The enzyme activity was measured using OPD (Sigma).

### Detection of intra cellular ROS (REACTIVE OXYGEN SPECIES)

The intracellular accumulation of ROS was determined using the fluorescent probe (2, 7, Dichloro Dihydro Fluorescein Diacetate) H2-DCFDA as described previously [56].

### Nitrite analysis and Arginase assay

Nitrite level in culture supernatants of BMDM and intracellular Arginase activity of both human monocytes and BMDM lysates were quantified as described elsewhere [57]. Arginase activity was measured in cell lysates. Briefly, cells were lysed using 50 µl of 0.1% Triton X-100. 5 µg Pepstatin and 5 µg aprotinin were used as protease inhibitors during lysis. This mixture was incubated for 30 min at room temperature. 50 µl of 10 mM MnCl2 and 50 mM Tris-HCl were added to lysed cells to activate the enzyme by heating for 10 min at 56°C. Then 25 µl of 0.5 M L-arginine, pH 9.7 was added and incubated at 37°C for 45 min. The reaction was stopped with 400 µl of H2SO4 (96%)/H3PO4 (85%)/H2O (1/3/7, v/v/v). 25 µl of α-isonitrosopropiophenone (dissolved in 100% ethanol) was added to the mixture followed by heating at 95°C for 45 min and urea concentration was measured at 540 nm.

### Docking studies

The X-ray structure of the extracellular domain of TLR4 (PDB code: 3FXI) [58] in complex with MD-2 is available. The X-ray structure of the TLR4-MD-2 complex (3FXI) was downloaded from the PDB data base. The TLR4 structure from this complex was extracted and used for docking with chtx. The chemical structure of chtx molecule was extracted from pubchem database (http://pubchem.ncbi.nlm.nih.gov). Structure of the chtx was retrieved into two-dimensional MDL/SDF format and three dimensional coordinates were generated using the ACCELRYS DS modelling 2.5 (Accelrys Inc. San Diego, CA 92121, USA) software suite. The missing hydrogen of the structure was fixed and subjected to energy minimization. All energy minimization were carried out using the conjugate gradient method of CHARM force field using the ACCELRYS DS modelling 2.5 (Accelrys Inc. San Diego, CA 92121, USA) software suite.

Docking studies were carried out using Genetic Optimization for Ligand Docking (GOLD) software, version 4.1.1 (Cambridge Crystallographic Data Centre, Cambridge, UK). The number of run was set to 100 in the standard default settings. The standard default settings, consisting of population size-100, selection pressure-1.1, niche size-2, migrate-10, cross over-95, number of operations-1,00,000 number of docking 10 were adopted for GOLD docking. For ligand- protein binding, 10 docking conformations (poses) were tested and the best GOLD score were considered for further analysis. The ligand showing maximum interactions with the protein were plotted using the program LIGPLOT. The active site was predicted by using Q-site finder [59].

### RNA isolation, reverse transcription and quantitative real time (QRT) PCR analysis

Total RNA was isolated from stimulated cells using RNAeasy columns from Qiagen, as per the manufacturer's instructions. 1 µg of total RNA from each sample was treated with DNAse I (Ambion Inc.). Synthesis of cDNA was performed by using First Strand Synthesis kit and the Superscript III Reverse Transcriptase (Invitrogen), according to the manufacturer's instructions. All real-time PCR experiments were performed in ABI prism 7900 HT sequence detection system (ABI) as described earlier [60]. The PCR conditions were as follows: 95°C for 10 min, 95°C for 15 sec, 58°C for 30 sec and 72°C for 30 sec for 40 cycles. The primers used for each gene are listed in (Table S 1). Primers were used at a concentration between 1 and 5 pmoles per reaction. All the reactions were analyzed using the software (SDS 2.3) provided with the instrument. The relative expression of the genes was calculated by using 2-ΔΔCt formula using GAPDH as a normalizer. The values reported are the mean of two biological replicates. The standard deviation from the mean is shown as error bars in each group.

### Statistical analysis

Statistical significance among experimental groups was analyzed by the unpaired Student's t-test using Graph pad prism software (Prism-5).

## Supporting Information

Figure S1 **A Glycoprotein fraction (AgW) purified from FAg binds to human monocytes.** Biotinylated FAg was passed through a WGA-sepharose affinity purification coloumn according to manufactures instructions. Both bound and unbound fractions were collected. Human PBMCs were incubated with both native, unbound (1^st^ elute) and bound (2^nd^ elute i.e. AgW) fractions followed by staining with Streptavidin FITC and the cells gated for monocytes were analyzed by FACS (**A**). N = 5. Bone marrow cells and human PBMCs were incubated with anti-mouse TLR4 PE (**B**) or anti- human TLR4 PE (**D**) respectively with and without FAg at 4°C for 30 minutes and analyzed by FACS. **B**- Representative overlaid histogram shows binding of anti-mouse TLR4 to CD 14+ve cells and competitive inhibition by FAg or AgW. **D**- Representative overlaid histogram shows binding of anti-human TLR4 to CD 14+ve cells and competitive inhibition by FAg. The monocyte or macrophage population binding to FAg was considered. % mean± SEM of five individual experiments performed with murine bone marrow cells and human PBMCs are also shown (Figures **S.**1**C** and **S.**1**E** respectively). * P<0.005, versus anti-TLR4 stained cells (Student's t-test). (**F**) Soluble TLR4 directly binds to the helminthic antigens ELISA plates were coated with (1 ug in PBS) extracts of *S.digitata*, AgW, *N.bracilliences*, *H.polygyrus*, *C.elegans*, mock BSA, PC-BSA, GlcNAc-BSA, Mannose-BSA or LPS. After blocking with 1% skimmed milk-PBS, lysates of mouse bone marrow cells were incubated for 2 hr at 37°C. The plates were thoroughly washed and were incubated with anti-mouse TLR4 and bound antibody was detected using peroxidase conjugated anti-rabbit IgG. The enzyme activity was measured using OPD. % mean ± SEM of two individual experiments are shown.
(TIF)

Figure S2 **Binding OF AgW to monocytes or macrophages is mediated through TLR4.** Bone marrow cells of BALB/c mice (**A,B,C**) or purified human PBMCs (**D,E,F**) were incubated with anti-mouse TLR4-PE and anti-human TLR4-PE respectively in the presence or absence of AgW at 4°C for 30 minutes, and CD 14^+^ cells analyzed by FACS. Representative dot plots show competitive inhibition of binding of anti-mouse antibodies (**A,B,C**) or anti-human antibodies (**D,E,F**) to TLR4.
(TIF)

Figure S3 **FAg interacts with TLR4 through carbohydrate residues.** **A,B** - Chitinase treated FAg or deglycosylated FAg fail to inhibit binding of Biotinylated FAg to human PBMCs. Human PBMCs were incubated with biotinylated FAg with and without chitinase treated FAg or sodium meta periodate treated deglycosylated FAg or cold unlabelled FAg at 4°C for 30 minutes followed by staining with Streptavidin-PE and cells gated for monocytes were analyzed by FACS. Representative overlaid histogram shows binding of FAg to cells inhibited by unlabelled FAg and absence of inhibition by chitinase treated FAg or sodium Meta periodate treated deglycosylated FAg. C-*In silico* analysis between chtx and TLR4. Three dimensional molecular docking between chtx and extra cellular domain of mouse TLR4 was performed using patch dock software demonstrating the interaction of chtx to the internal pocket of extra cellular domain of TLR4.
(TIF)

Figure S4 **FAg or chtx induce expression of Ym-1 and Arginase-1 in both** ***in vitro*** **and** ***in vivo*** **.** BMDM of BALB/c mice were treated with FAg or chtx for 48 hrs. and were analysed for expression of Ym-1(**A–D**, isotype control, basal, FAg and chtx respectively) and Arginase-1 (**E–H** isotype control, basal, FAg and chtx respectively) by intracellular staining. Representative dot plots show FAg or chtx induced expression of Ym-1 and Arginase-1 in CD14+ve mouse macrophages. **I,J:** BALAB/c mice were injected intraperitoneally with LPS (15 mg/Kg body weight) or chtx (250 µg/animal) and after 90 mins peritoneal cells were harvested, washed and stained for Ym-1 or Arginase-1 and analyzed for expression in CD14+ve cells and is shown as histograms.
(TIF)

Figure S5 **Monocytes of subjects with active filarial infection are less responsive to LPS induced signaling.** (**A,B,C**) PBMCs of subjects with active filarial infection and endemic controls were cultured in sterile Dulbecco's modified eagles medium containing 10% autologus plasma in humidified atmospheric condition (5% CO_2_, 80% humidity). After 8–10 hrs incubation at 370C non adherent cells were removed by washing with sterile medium. The remaining adherent cells were stimulated with different concentrations of LPS for 48 hrs. The culture supernatants were analyzed for TNF-α (**A**), IL-1β (**B**) and and IL-6 (**C**). N = 6 for CFA−ve and 5 for CFA+ve. * P<0.001, versus CFA−ve cells (Student's t-test).
(TIF)

Figure S6 **Test of purity of adherent cells.** Washed mouse bone marrow cells in DMEM were seeded in 24 well plates at 0.5×10^6^ cells/well and incubated for 8–10 hrs at 37°C. Non adherent cells were removed by washing with sterile DMEM and the adherent cells were stained for CD14 (Figure **S.6 B**) along with isotype controls (Figure **S.6 A**).
(TIF)

Table S1 **Real time PCR was performed for the following genes IL-1β, IL-6, TNF-α, IL-12 and iNOS and GAPDH control using RNA purified from BMDM stimulated with LPS or Chitohexaose.** Details of primers used for quantitative real time (QRT) PCR study are shown in Table S1.
(DOC)
