# Supplementary figures and images for: Chitohexaose Activates Macrophages by Alternate Pathway through TLR4 and Blocks Endotoxemia
Source: PLoS Pathog. 2012 May 24;8(5):e1002717. doi: 10.1371/journal.ppat.1002717 (PMC3359989; doi:10.1371/journal.ppat.1002717)

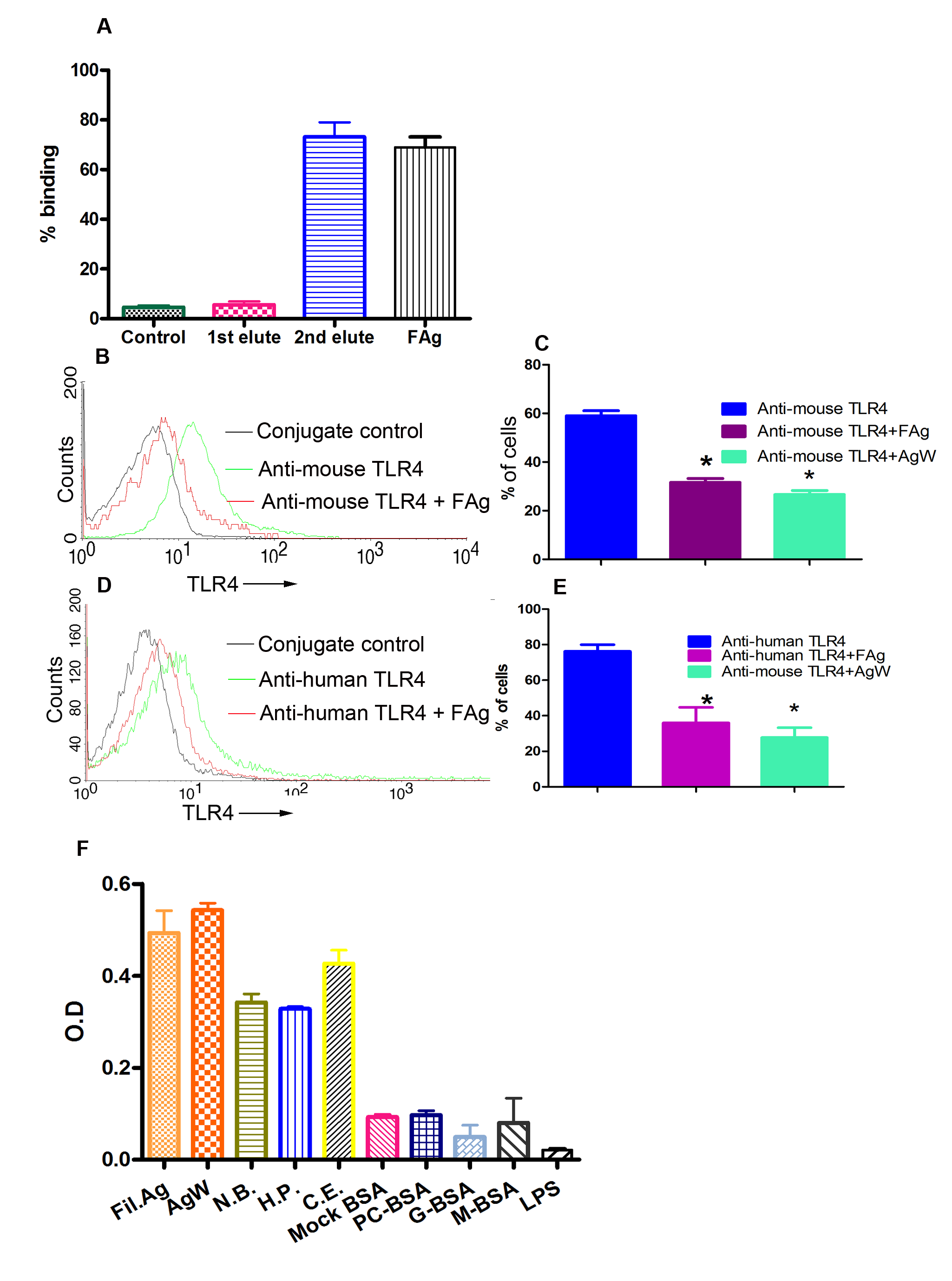

Supplement: Figure S1 — A Glycoprotein fraction (AgW) purified from FAg binds to human monocytes. Biotinylated FAg was passed through a WGA-sepharose affinity purification coloumn according to manufactures instructions. Both bound and unbound fractions were collected. Human PBMCs were incubated with both native, unbound (1st elute) and bound (2nd elute i.e. AgW) fractions followed by staining with Streptavidin FITC and the cells gated for monocytes were analyzed by FACS (A). N = 5. Bone marrow cells and human PBMCs were incubated with anti-mouse TLR4 PE (B) or anti- human TLR4 PE (D) respectively with and without FAg at 4°C for 30 minutes and analyzed by FACS. B- Representative overlaid histogram shows binding of anti-mouse TLR4 to CD 14+ve cells and competitive inhibition by FAg or AgW. D- Representative overlaid histogram shows binding of anti-human TLR4 to CD 14+ve cells and competitive inhibition by FAg. The monocyte or macrophage population binding to FAg was considered. % mean± SEM of five individual experiments performed with murine bone marrow cells and human PBMCs are also shown (Figures S.1C and S.1E respectively). * P<0.005, versus anti-TLR4 stained cells (Student's t-test). (F) Soluble TLR4 directly binds to the helminthic antigens ELISA plates were coated with (1 ug in PBS) extracts of S.digitata, AgW, N.bracilliences, H.polygyrus, C.elegans, mock BSA, PC-BSA, GlcNAc-BSA, Mannose-BSA or LPS. After blocking with 1% skimmed milk-PBS, lysates of mouse bone marrow cells were incubated for 2 hr at 37°C. The plates were thoroughly washed and were incubated with anti-mouse TLR4 and bound antibody was detected using peroxidase conjugated anti-rabbit IgG. The enzyme activity was measured using OPD. % mean ± SEM of two individual experiments are shown. (TIF) [file ppat.1002717.s001.tif]

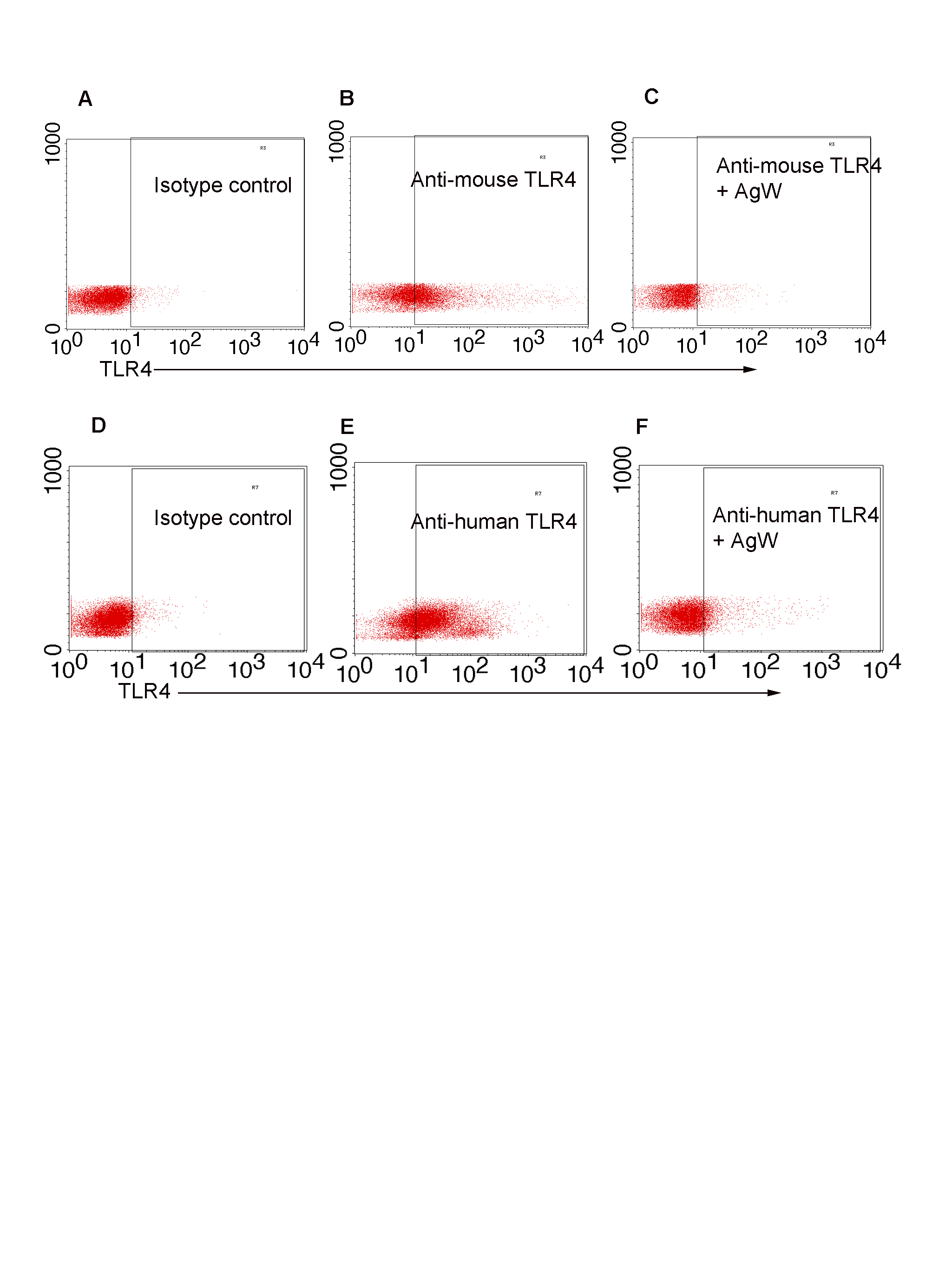

Supplement: Figure S2 — Binding OF AgW to monocytes or macrophages is mediated through TLR4. Bone marrow cells of BALB/c mice (A,B,C) or purified human PBMCs (D,E,F) were incubated with anti-mouse TLR4-PE and anti-human TLR4-PE respectively in the presence or absence of AgW at 4°C for 30 minutes, and CD 14+ cells analyzed by FACS. Representative dot plots show competitive inhibition of binding of anti-mouse antibodies (A,B,C) or anti-human antibodies (D,E,F) to TLR4. (TIF) [file ppat.1002717.s002.tif]

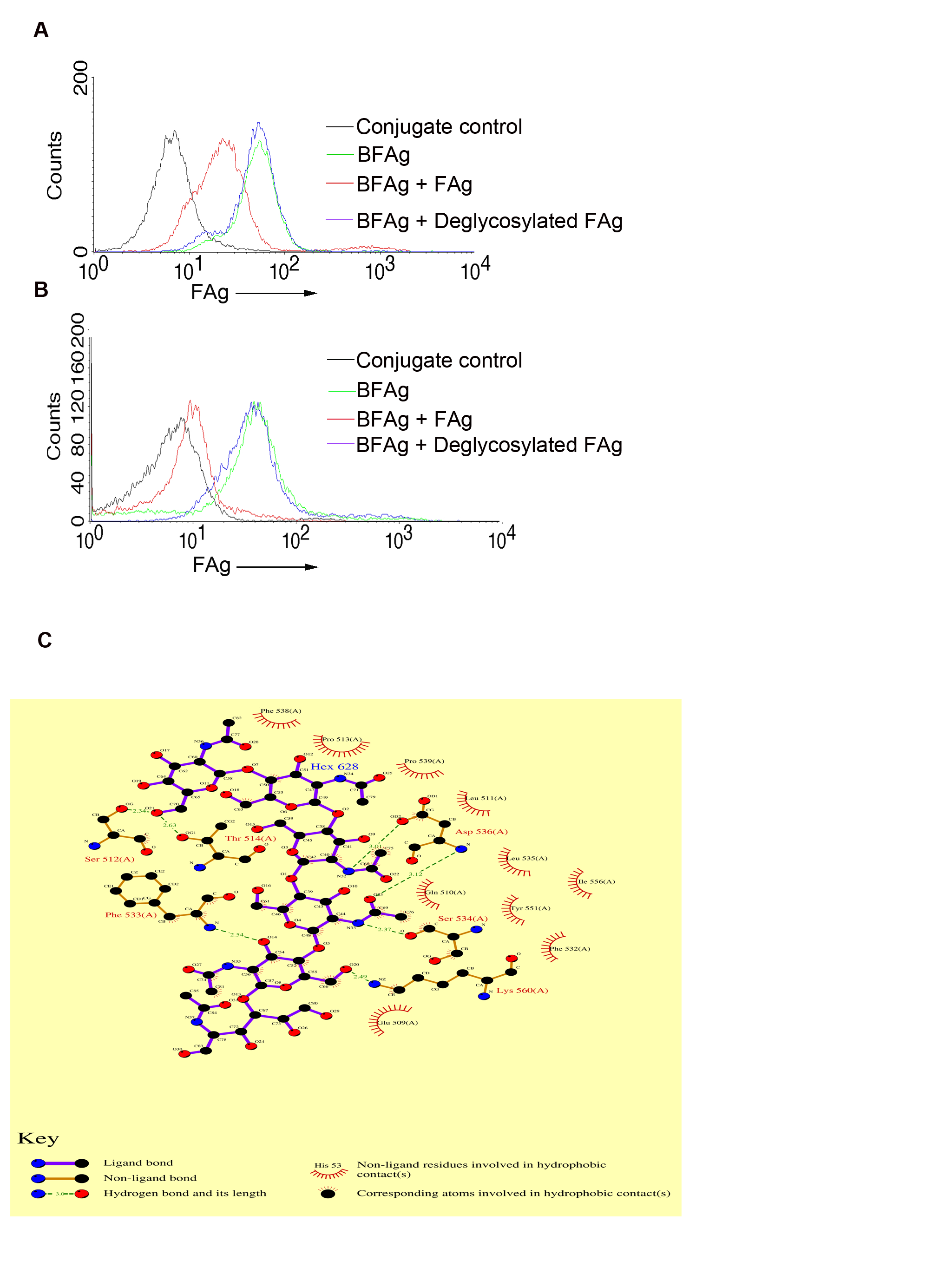

Supplement: Figure S3 — FAg interacts with TLR4 through carbohydrate residues. A,B - Chitinase treated FAg or deglycosylated FAg fail to inhibit binding of Biotinylated FAg to human PBMCs. Human PBMCs were incubated with biotinylated FAg with and without chitinase treated FAg or sodium meta periodate treated deglycosylated FAg or cold unlabelled FAg at 4°C for 30 minutes followed by staining with Streptavidin-PE and cells gated for monocytes were analyzed by FACS. Representative overlaid histogram shows binding of FAg to cells inhibited by unlabelled FAg and absence of inhibition by chitinase treated FAg or sodium Meta periodate treated deglycosylated FAg. C-In silico analysis between chtx and TLR4. Three dimensional molecular docking between chtx and extra cellular domain of mouse TLR4 was performed using patch dock software demonstrating the interaction of chtx to the internal pocket of extra cellular domain of TLR4. (TIF) [file ppat.1002717.s003.tif]

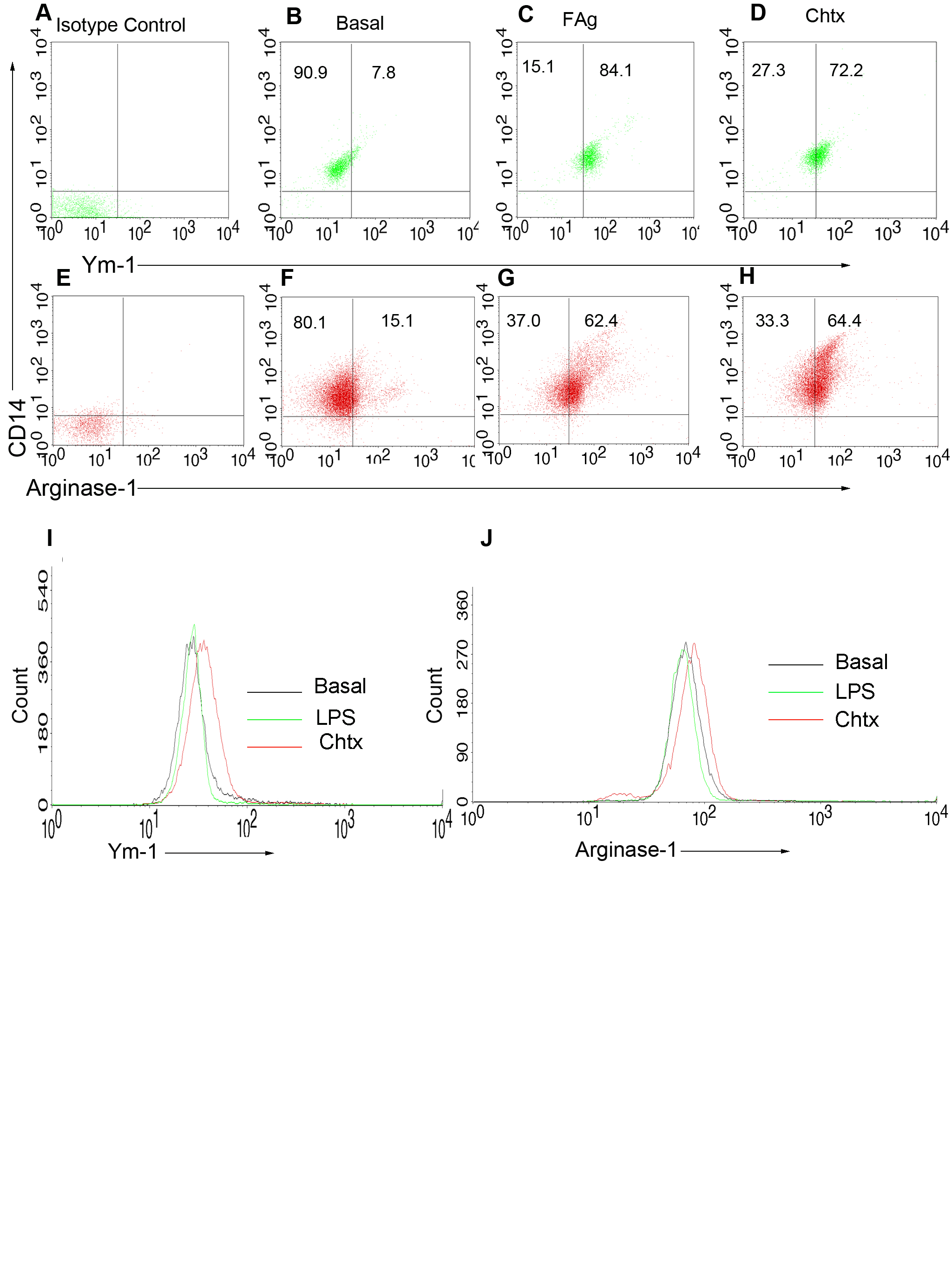

Supplement: Figure S4 — FAg or chtx induce expression of Ym-1 and Arginase-1 in both in vitro and in vivo . BMDM of BALB/c mice were treated with FAg or chtx for 48 hrs. and were analysed for expression of Ym-1(A–D, isotype control, basal, FAg and chtx respectively) and Arginase-1 (E–H isotype control, basal, FAg and chtx respectively) by intracellular staining. Representative dot plots show FAg or chtx induced expression of Ym-1 and Arginase-1 in CD14+ve mouse macrophages. I,J: BALAB/c mice were injected intraperitoneally with LPS (15 mg/Kg body weight) or chtx (250 µg/animal) and after 90 mins peritoneal cells were harvested, washed and stained for Ym-1 or Arginase-1 and analyzed for expression in CD14+ve cells and is shown as histograms. (TIF) [file ppat.1002717.s004.tif]

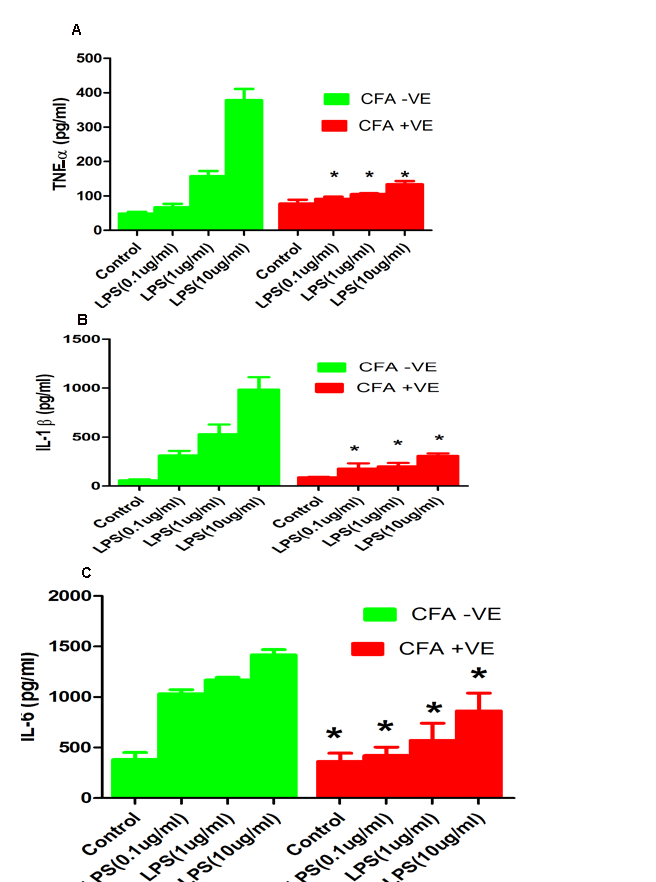

Supplement: Figure S5 — Monocytes of subjects with active filarial infection are less responsive to LPS induced signaling. (A,B,C) PBMCs of subjects with active filarial infection and endemic controls were cultured in sterile Dulbecco's modified eagles medium containing 10% autologus plasma in humidified atmospheric condition (5% CO2, 80% humidity). After 8–10 hrs incubation at 370C non adherent cells were removed by washing with sterile medium. The remaining adherent cells were stimulated with different concentrations of LPS for 48 hrs. The culture supernatants were analyzed for TNF-α (A), IL-1β (B) and and IL-6 (C). N = 6 for CFA−ve and 5 for CFA+ve. * P<0.001, versus CFA−ve cells (Student's t-test). (TIF) [file ppat.1002717.s005.tif]

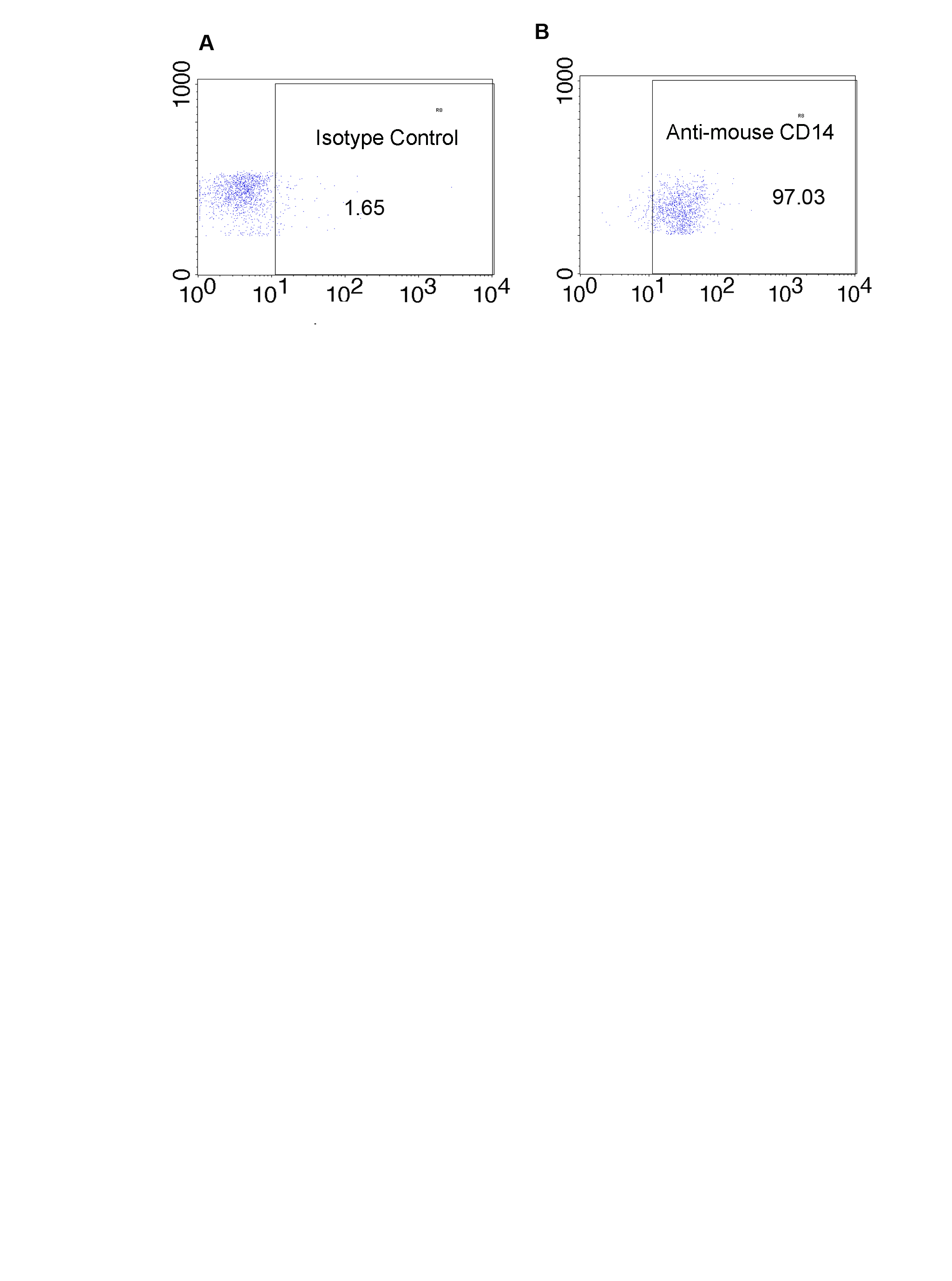

Supplement: Figure S6 — Test of purity of adherent cells. Washed mouse bone marrow cells in DMEM were seeded in 24 well plates at 0.5×10^6^ cells/well and incubated for 8–10 hrs at 37°C. Non adherent cells were removed by washing with sterile DMEM and the adherent cells were stained for CD14 (Figure S.6 B) along with isotype controls (Figure S.6 A). (TIF) [file ppat.1002717.s006.tif]
