# Supplementary material for: Chitohexaose Activates Macrophages by Alternate Pathway through TLR4 and Blocks Endotoxemia
Source: PLoS Pathog. 2012 May 24;8(5):e1002717. doi: 10.1371/journal.ppat.1002717 (PMC3359989; doi:10.1371/journal.ppat.1002717)
Supplement: Table S1 — Real time PCR was performed for the following genes IL-1β, IL-6, TNF-α, IL-12 and iNOS and GAPDH control using RNA purified from BMDM stimulated with LPS or Chitohexaose. Details of primers used for quantitative real time (QRT) PCR study are shown in Table S1. (DOC) [file ppat.1002717.s007.doc]

|  | Forward primer (5’-3’) | Reverse primer (5’-3’) |
| --- | --- | --- |
| GAPDH | ATG GCC TTC CGT GTT CCT A | TGA AGT CGC AGG AGA CAA CCT |
| IL-1β | TTGACGGACCCCAAAAGATG | AGAAGGTGCTCATGTCCTCA |
| IL-6 | GTTCTCTGGGAAATCGTGGA | TGTACTCCAGGTAGCTATGG |
| TNF-α | CCGTCAGCCGATTTGCTATCTCA | CAAAGTAGACCTGCCCGGACT |
| IL-12 | GTC CGA TCC TAG GAT GCA AC | ATA CAT TAG CTC CCT GGC TCT G |
| iNOS | TGT CCT ACA CCA CAC CAA ACT G | CTC CAA TCT CTG CCT ATC CGT C |

Supporting information : Table 1: Details of primers used for Real time PCR
